# Modulating intrinsic functional connectivity with visual cortex using low‐frequency repetitive transcranial magnetic stimulation

**DOI:** 10.1002/brb3.2491

**Published:** 2022-01-20

**Authors:** Sara A. Rafique, Jennifer K. E. Steeves

**Affiliations:** ^1^ Department of Psychology and Centre for Vision Research York University Toronto Canada

**Keywords:** accelerated rTMS, functional connectivity, GABA, glutamate, repetitive TMS, resting‐state fMRI, visual cortex

## Abstract

**Introduction:**

Intrinsic network connectivity becomes altered in pathophysiology. Noninvasive brain stimulation can modulate pathological functional networks in an attempt to restore the inherent response. To determine its usefulness for visual‐related disorders, we developed procedures investigating repetitive transcranial magnetic stimulation (rTMS) protocols targeting the visual cortex on modulating connectivity associated with the visual network and default mode network (DMN).

**Methods:**

We compared two low‐frequency (1 Hz) rTMS protocols to the visual cortex (V1)—a single 20 min session and five successive 20 min sessions (accelerated/within‐session rTMS)—using multi‐echo resting‐state functional magnetic resonance whole‐brain imaging and resting‐state functional connectivity (rsFC). We also explored the relationship between rsFC and rTMS‐induced changes in key inhibitory and excitatory neurotransmitters, γ‐aminobutyric acid (GABA) and glutamate. GABA (GABA+) and glutamate (Glx) concentrations were measured in vivo using magnetic resonance spectroscopy.

**Results:**

Acute disruption with a single rTMS session caused widespread connectivity reconfiguration with nodes of interest. Changes were not evident immediately post‐rTMS but were observed at 1 h post‐rTMS. Accelerated sessions resulted in weak alterations in connectivity, producing a relatively homeostatic response. Changes in GABA+ and Glx concentrations with network connectivity were dependent on the rTMS protocol.

**Conclusions:**

This proof‐of‐concept study offers new perspectives to assess stimulation‐induced neural processes involved in intrinsic functional connectivity and the potential for rTMS to modulate nodes interconnected with the visual cortex. The differential effects of single‐session and accelerated rTMS on physiological markers are crucial for furthering the advancement of treatment modalities in visual cortex related disorders.

## Graphical abstract

Acute disruption to V1 with a single session of 1 Hz repetitive transcranial magnetic stimulation (rTMS) reconfigures intrinsic resting‐state functional connectivity (rsFC) to regions within and outside the visual and default mode networks. Accelerated 1 Hz rTMS sessions to V1 maintains a homeostatic response on rsFC. Changes in rsFC were influenced by rTMS‐induced changes in GABA and glutamate concentrations.

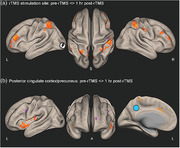

## 1 INTRODUCTION

Noninvasive brain stimulation allows modulation of cortical networks through plasticity and provides a valuable tool to investigate and manipulate neural mechanisms required for promoting recovery of brain function in neurological and psychiatric disorders. Repetitive transcranial magnetic stimulation (rTMS), a prevalent noninvasive brain stimulation technique, can be used to induce longer‐lasting neuroplastic changes in a variety of cortical and subcortical regions in the order of minutes to hours, and even months (Dunner et al., 2014; Liepert et al., 2000; U. Ziemann et al., 2008). Stimulation influences neuronal properties of the stimulated region, which is typically an accessible network node toward the surface of the cortex. What is less understood is the stimulation response that propagates transynaptically to functionally interconnected nodes. A variety of rTMS protocols modulate beyond the stimulation site to alter extended functional networks (Rafique et al., 2015; van der Werf et al., 2010; Watanabe et al., 2014). Resting‐state functional magnetic resonance imaging (rsfMRI) enables investigation of intrinsic network connectivity, and more broadly can also be used as a biomarker to explore the effects of noninvasive brain stimulation on connectivity. Resting‐state functional connectivity (rsFC) provides a measure of synchronous fluctuations in blood oxygen level‐dependent (BOLD) signal (a surrogate measure of neuronal activity; Logothetis et al., 2001) among regions to determine functional connectivity between interconnected nodes within networks. As such, rsFC provides insight into the functional organization of brain networks and baseline neural processing at rest (Fox & Raichle, 2007). Altered rsFC is increasingly used as a biomarker in many neurological and psychiatric disorders that show variability in the strength of functional coupling within distinct networks, for example, depression, Alzheimer's disease, and schizophrenia (for reviews, see M. Greicius, 2008; Mulders et al., 2015; Whitfield‐Gabrieli & Ford, 2012). The combination of rTMS and rsFC offers a promising technique to both identify and modulate pathological network interactions for perceptual, behavioral, and/or neurochemical gains in conditions associated with altered network connectivity (Fox et al., 2012; Grefkes et al., 2010; Strafella et al., 2003).

The combined application of rsFC and rTMS in visual disorders has, however, received little attention. Altered communication is observed in visual hallucinations associated with visual loss that stem from disorganized functional activity in interconnected cortical and subcortical networks (ffytche et al., 1998; Rafique et al., 2016). Widespread alterations in rsFC are observed in other cases of vision disorders such as amblyopia, suggesting that deficits are related to abnormal neural connections across networks (Wang et al., 2014). rTMS may provide an alternative therapeutic tool for visual disorders that are nonresponsive to other treatment modalities (Merabet et al., 2003; Rafique et al., 2016; Thompson et al., 2008). While rTMS can alter functional network connectivity, the effects can be unpredictable, unstable, and short‐lasting, particularly in pathophysiology (Maeda et al., 2000; Ridding & Ziemann, 2010), thus limiting its usefulness. For noninvasive brain stimulation to be useful in re‐establishing intrinsic network connectivity in visual disorders, data are needed from both healthy and patient populations. A reference model of expected effects in healthy controls can elucidate underlying mechanisms of rTMS from which therapeutic protocols for visual‐related disorders can be developed, and patient data can be compared. Knowledge of neurophysiological responses to different stimulation protocols in healthy individuals can then guide selective modification of connectivity between specific brain regions in a controlled manner.

We explored the efficacy of low‐frequency (1 Hz) rTMS to the visual cortex on modulating associated intrinsic functional network connectivity and investigated whether changes persisted beyond the immediate post‐rTMS measured effects. We employed shortened schedules of two common stimulation protocols that are used in nonvisual disorders: a single session of rTMS applied over consecutive days for weeks/months, and accelerated sessions (also termed within‐session rTMS; multiple sessions within one day) applied over consecutive days for a shorter period. Previous research using accelerated rTMS in patient populations (in nonvisual disorders) suggests that despite the reduced number of stimulation days compared with single‐session rTMS, the increased stimulation doses within a single day produce augmented effects compared to single sessions over consecutive days (for a review, see Goldsworthy et al., 2015). Accelerated rTMS had not previously been applied to the visual cortex or visual disorders. Our shortened schedule of these two common protocols consisted of (1) a single 20 min session and (2) five accelerated 20 min sessions. We considered the direct effect of rTMS to V1 (a key node in the visual processing network; Beckmann et al., 2005; Yeo et al., 2011) and the indirect effect on the default mode network (DMN) on whole‐brain connectivity. We chose to investigate the indirect effects on the DMN since DMN dysfunction is suggested to arise in disorders affecting visual processing (Lewis et al., 2014). The DMN consists of regions that show increased levels of activity during rest and are engaged in spontaneous and self‐generated mental activity in the absence of external attentional demands (Gusnard et al., 2001). Additionally, the DMN is of importance when considering modulating networks since it is functionally and structurally interconnected with a considerable number of cortical and subcortical regions (Buckner et al., 2011; Hagmann et al., 2008; Margulies et al., 2009). Therefore, significant modulation of the DMN following stimulation may have substantial implications for cognitive and behavioral performance. rsfMRI data were processed using multi‐echo independent components analysis (ME‐ICA). ME‐ICA uses ME fMRI acquisition and echo time (TE) dependency of resting‐state BOLD signal to denoise artefactual fluctuations more effectively than other approaches (e.g., physiological noise modeling, band‐pass filtering) and improves temporal signal‐to‐noise ratio (Kundu et al., 2012). By attenuating non‐BOLD‐related noise, ME‐ICA addresses the issue of low statistical power in fMRI studies, correspondingly improving the sensitivity and statistical power of fMRI (Kundu et al., 2012). The enhanced sensitivity of ME‐ICA facilitates the detection of smaller and more subtle effects in brain regions that are methodologically fraught by other denoising methods (for a review, see Kundu et al., 2017; Lombardo et al., 2016). Finally, to provide a more extensive investigation of rTMS effects, we considered the effect of changes in key inhibitory and excitatory neurotransmitters, γ‐aminobutyric acid (GABA), and glutamate, respectively, on impacting intrinsic functional connectivity modulation. The coordination between GABAergic interneurons and glutamatergic neurons directly impacts the BOLD signal (Logothetis, 2008; Magistretti & Pellerin, 1999; A. J. Smith et al., 2002). However, the neurochemical basis for variability in rsFC remains poorly understood and is to some extent related to GABAergic and glutamatergic systems. Assessing the neurochemical underpinnings of network connectivity will provide a better understanding of healthy and pathological neurophysiological responses associated with GABAergic/glutamatergic systems, with translation to therapeutic protocols of noninvasive brain stimulation. We had previously investigated the effects of single‐session and accelerated rTMS on visual cortex neurotransmitters and basic visual function, as well as longer‐term monitoring of potential adverse effects (Rafique & Steeves, 2020).

Overall, the motivations of this study were to (1) provide detailed methods and considerations for combining a variety of neuromodulation and neuroimaging techniques in an interdisciplinary approach for a more comprehensive investigation of rTMS effects at the visual cortex and associated networks, (2) investigate a rTMS protocol previously not employed at the visual cortex, namely accelerated rTMS, and (3) provide important proof‐of‐concept data to inform future clinical trials to treat visual disorders.

## 2 METHODS

This study was approved by York University's Office of Research Ethics. All individuals gave informed written consent.

### 2.1 Participants

Sixteen participants took part in the study (mean_age_ ± SEM = 25.15 ± 1.21 years; 10 males/six females). All participants were right‐handed, with normal or corrected‐to‐normal vision (>0.04 logMAR), and had no known contraindications to TMS and MRI. Participants had no known underlying medical conditions, no history of neurological or psychological disorders, and were not taking any medications at the time of the study. Due to interactions with TMS mechanisms, we recruited participants with no history of frequent or chronic migraines (Bohotin et al., 2002). All participants had also taken part in a magnetic resonance spectroscopy (MRS) study to quantify changes in visual cortex GABA and glutamate concentrations during the same experimental procedure as the present study (Rafique & Steeves, 2020). Complete details on exclusion criteria are provided in our previous MRS study.

### 2.2 Experimental design overview

**FIGURE 1:**
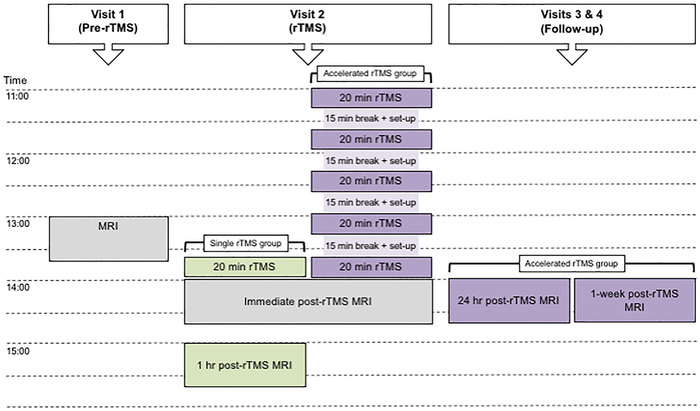
Overview of the experimental procedure. All participants took part in procedures in gray, participants undergoing a single rTMS session took part in procedures in green, and participants who underwent accelerated rTMS took part in procedures in purple. Abbreviations: MRI, magnetic resonance imaging; rTMS, repetitive transcranial magnetic stimulation. Image adapted from Rafique & Steeves, 2020

We used a parallel‐group design. Participants initially underwent pre‐rTMS (baseline) ME rsfMRI. In a separate follow‐up visit, participants received offline 1 Hz rTMS to the visual cortex (V1) at their phosphene threshold (PT), either in (1) a single 20 min session of rTMS, or (2) five accelerated 20 min sessions of rTMS (separated by intervals of ∼15 min). ME rsfMRI was repeated immediately following rTMS in both groups and was repeated at (1) 1 h post‐rTMS in the single rTMS group and (2) 24 h and 1 week post‐rTMS in the accelerated rTMS group. See Figure 1 for an overview.

### 2.3 Magnetic resonance imaging acquisition

ME rsfMRI and anatomical data were acquired with a 3T Siemens Magnetom Tim Trio magnetic resonance scanner with a 32‐channel high‐resolution brain array coil (Siemens, Erlangen, Germany). Head motion was minimized using soft pads surrounding participants’ heads. Imaging was acquired at rest in a dark room, and participants were instructed to keep their eyes closed and not to think of anything in particular throughout.

ME rsfMRI data were acquired first to capture immediate post‐rTMS effects using whole‐brain ME echo‐planar imaging with a T2*‐weighted sequence (43 contiguous axial slices; in‐plane resolution = 3.4 × 3.4 mm; slice thickness = 3.0 mm; imaging matrix = 64 × 64; repetition time (TR) = 3000 ms; TE = 14, 30, 46 ms; flip angle = 83^°^; field of view (FoV) = 216 mm; acquisition time = ∼10 min). Anatomical images were acquired after rsfMRI with a T1 magnetization‐prepared rapid gradient echo imaging sequence (number of slices = 192; in‐plane resolution = 1 × 1 mm; slice thickness = 1.0 mm; imaging matrix = 256 × 256; TR = 2300 ms; TE = 2.62 ms; inversion time = 900 ms; flip angle = 9°; FoV = 256 mm; acquisition time = ∼5 min).

### 2.4 Transcranial magnetic stimulation

**FIGURE 2:**
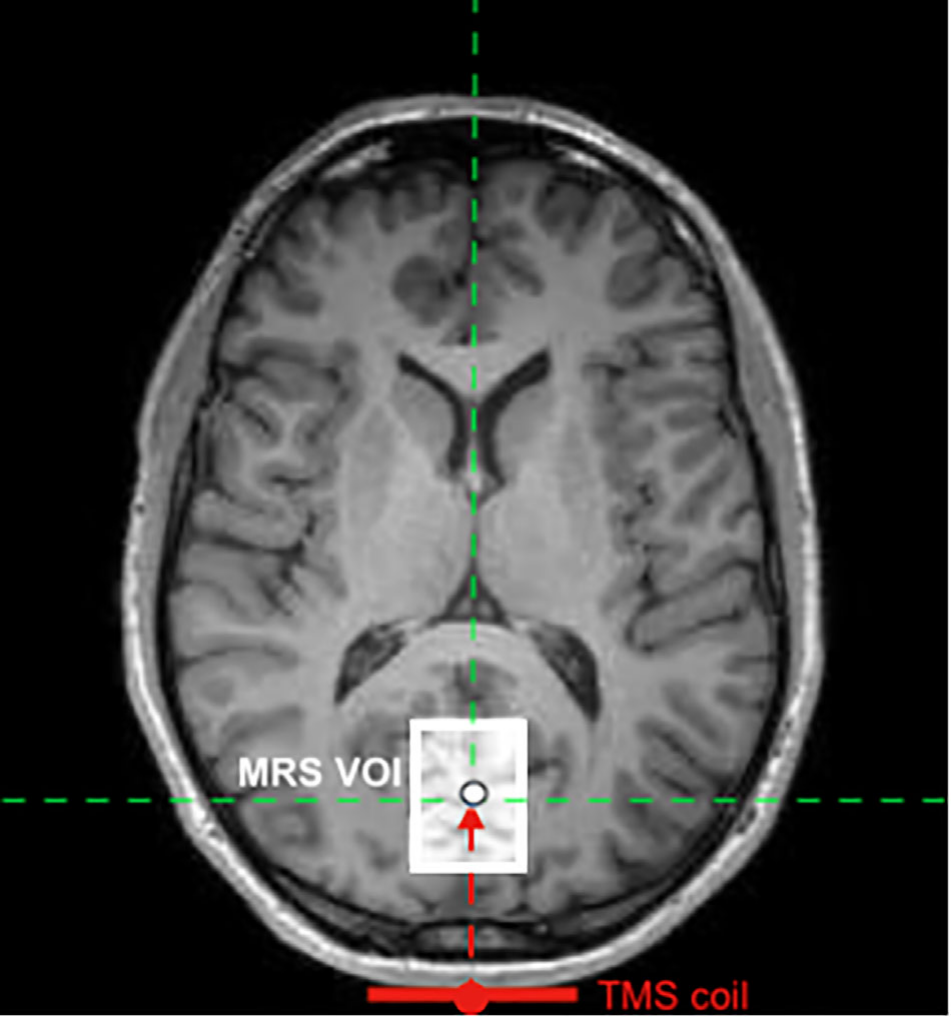
Positioning of the TMS target site and coil with relation to the MRS VOI. The target stimulation site (black circle) was positioned to coincide with the center of the MRS VOI (white box) for each participant individually (mean_depth_ ± SEM = 38.631 ± 0.74 mm). The crosshair (junction between red and green dashed lines) shows the center of the MRS VOI and accordingly the rTMS target site. The red arrow demonstrates the direction of stimulation toward the target site from the TMS coil. The VOI was positioned as far back within the posterior region of the occipital pole, centered on the calcarine sulcus and aligned alongside the cerebellar tentorium while avoiding any nonbrain tissue. Abbreviations: MRS, magnetic resonance spectroscopy; TMS, transcranial magnetic stimulation; rTMS, repetitive TMS; VOI, volume‐of‐interest

Anatomical MR images were reconstructed to three‐dimensional cortical surfaces, and individual stimulation sites were mapped to their corresponding reconstructed surface using Brainsight software (Rogue Research, Montreal, QC, Canada). Participant target stimulation sites in the visual cortex were based on our MRS study and corresponded to the center of the MRS volume‐of‐interest (see Figure 2 for an overview) from which GABA (represented by GABA+, the combined concentration of GABA and macromolecules) and glutamate (assessed via Glx, a composite of glutamate and glutamine) concentrations were extracted (for a full description, see Rafique & Steeves, 2020). This enabled analysis of a direct relationship between changes in neurotransmitter metabolites and rsFC following the two rTMS protocols. Anatomical images in native space were coregistered to standardized Montreal Neurological Institute (MNI) coordinate space within Brainsight using a linear transformation (translation, rotation, and scaling). Images were converted to MNI space to obtain individual standardized rTMS target site coordinates (see Table S1) that would be used for rsfMRI V1 seed analyses. Brainsight MNI space images were not used for further analyses. Positioning of the coil with respect to the participant's head and the stimulation site was visualized in real‐time using a Polaris infrared image‐guided tracking system (Northern Digital Instruments, Kitchener, ON, Canada) to ensure accurate and targeted stimulation throughout. Participants were seated in a comfortable position with an adjustable chin rest to limit head movement and provided with earplugs to prevent changes in auditory thresholds during rTMS (Rossi et al., 2009). A Magstim Rapid^2^ Stimulator and a 70 mm diameter figure‐of‐eight coil (Magstim, Whitland, Wales, UK) were used to deliver the stimulation pulses. The coil was held parallel to the midline with the handle pointing downward, and the coil center was held tangential to the surface of the skull to minimize coil–cortex distance and thereby maximize the TMS effect (Ulmer & Jansen, 2010). rTMS was delivered to the stimulation site at the participant's PT to minimize interindividual variability in visual cortical excitability thresholds (Stewart et al., 2001). Full details on obtaining PT are described in our previous study(see ‘‘Methods’’ in Rafique & Steeves, 2020). Individual PTs are provided in Table S1. Participants underwent offline 1 Hz rTMS (100% PT) at rest either in (1) a single 20 min session (1200 pulses; *n *= 8, 4 male) or (2) five accelerated 20 min sessions separated by ∼15 min within a single day (each session 1200 pulses, total 6000 pulses; *n *= 8, 6 male). We chose to investigate low‐frequency rTMS based on evidence that 1 Hz rTMS to the visual cortex induces dishabituation of electrophysiological responses (visual evoked potentials), whereas 10 Hz (high‐frequency) rTMS of comparable pulses has no significant effect (Bohotin et al., 2002; Fumal et al., 2003). Moreover, 15−20 min daily sessions of 1 Hz rTMS to the visual cortex for five consecutive days produce an accumulative effect in dishabituation (Fumal et al., 2006) and modulating visual cortical activity (Rafique et al., 2016). We opted for 20 min of stimulation since it is more effective than shorter application times and reduces interindividual variability (Aydin‐Abidin et al., 2006). Intervals of 10−20 min in accelerated stimulation produce longer‐lasting effects (for a review, see Goldsworthy et al., 2015) compared with shorter intervals, for example, 3 min (Monte‐Silva et al., 2010) or 5 min (Bastani & Jaberzadeh, 2014).

### 2.5 Experimental procedure

Visit 1 (baseline): participants initially underwent pre‐rTMS ME rsfMRI commencing at ∼13:00. PTs were determined after pre‐rTMS rsfMRI usually on the same day, or at a similar time on a different day. Visit 2 (rTMS): on a different day, participants underwent rTMS to the visual cortex, which commenced at ∼13:40 for participants in the single rTMS group, and commenced at ∼11:00 for participants in the accelerated rTMS group. These times were chosen so that rTMS would cease at the same time of the day irrespective of group, and immediate post‐rTMS rsfMRI would be performed at ∼14:00 in both groups to minimize diurnal confounds. Following the completion of the rTMS protocol, participants were immediately transferred into the MRI scanner and acquisition commenced within 5 min of rTMS ending. Participants in the single rTMS group underwent further rsfMRI 1 h post‐rTMS at ∼15:00. rTMS visits were scheduled at least 4 days following PT measures in both groups to prevent any lingering TMS effects from PT measurement interacting with the rTMS protocol. Visits 3 and 4 (follow‐up for accelerated rTMS group only): participants in the accelerated rTMS group underwent further rsfMRI at 24 h and 1 week post‐rTMS, both at ∼14:00. We did not perform further follow‐up visits in the single rTMS group based on previous research demonstrating aftereffects following ∼15−20 min 1 Hz rTMS to the occipital cortex recover within 20−40 min (for a review, see Thut & Pasucal‐Leone, 2010). We did not perform MRI 1 h post‐rTMS in the accelerated rTMS group because of fatigue following a long protocol (∼5 h), and since effects were expected to persist for > 24 h (for a review, see Goldsworthy et al., 2015). MRS data were collected during the same MRI acquisition as rsfMRI in order to support any correlation between changes in rsFC and GABA+ and Glx concentrations following rTMS.

### 2.6 Data analyses

#### 2.6.1 Preprocessing and denoising

ME rsfMRI data were initially preprocessed and denoised with the following steps using the AFNI integrated ME‐ICA pipeline (v3.2.2; http://afni.nimh.nih.gov; Kundu et al., 2013, 2012). The first five TRs were removed to achieve steady‐state equilibration. MR images were skull‐stripped and intensity‐normalized. Images were deobliqued (3dWarp), slice‐timing corrected, axialized (3daxialize), and despiked (3dDespike). Motion correction parameters were estimated for each time point by aligning the middle TE (30 ms) images to the corresponding first time point image using a rigid body (six parameters) alignment procedure. Functional and anatomical images were coregistered by registering the skull‐stripped middle TE image from the first time point to the skull‐stripped anatomical image using affine (12 parameters) alignment with the local Pearson correlation and T2* weights (3dSkullStrip, 3dAllineate). Motion correction and anatomical coregistration parameters were then applied in one step (3dAllineate). Functional and anatomical images additionally underwent nonlinear warping to standard MNI space (3dQWarp; MNI Colin27, 1 × 1 × 1 mm). The three TEs provide different BOLD contrasts and are combined to create an optimal combination of contrast specific to each voxel, therefore producing a more homogenous contrast‐to‐noise ratio compared with single‐echo fMRI (Kundu et al., 2015). The concatenated optimally combined functional data underwent principal component analysis (PCA) to distinguish the BOLD signal of high and low variance components from noise. Denoising in ME‐ICA is achieved with FastICA, which decomposes and classifies the retained components from PCA into BOLD signal and non‐BOLD noise and effectively removes noise components using linear regression, including motion, physiological and scanner artefacts, for example, draining veins and in‐plane acceleration (Evans et al., 2015; Kundu et al., 2017). Using non‐BOLD component time courses as noise regressors greatly improves seed‐based correlation mapping by minimizing the influence of high‐ and low‐frequency non‐BOLD fluctuations (Kundu et al., 2012). ME‐ICA denoising retains thermal noise and low variance ICs with high degrees of freedom, thereby increasing the temporal signal‐to‐noise ratio (Kundu et al., 2015) and sensitivity for determining significant effects (Kundu et al., 2015, 2017).

In CONN: functional connectivity toolbox (v17.f; http://www.nitrc.org/projects/conn; Whitfield‐Gabrieli & Nieto‐Castanon, 2012), a Matlab‐based cross‐platform, ME‐ICA denoised time series were spatially smoothed using a 6 mm full width at half maximum Gaussian kernel. Each participant's MNI space anatomical images from each visit were segmented into cerebrospinal fluid, and gray and white matter using SPM8 (Statistical Parametric Mapping; Wellcome Centre for Human Neuroimaging, London, UK; http://www.fil.ion.ucl.ac.uk/spm/) unified segmentation procedure (Ashburner & Friston, 2005) in CONN. The BOLD signal from white matter and cerebrospinal fluid masks were identified using component‐based noise correction (CompCor; Behzadi et al., 2007), and associated residual confounding effects were linearly regressed with PCA (five components each with no additional temporal expansion derivative terms; Chai et al, 2012) to improve (center) the distribution of connectivity values of the data. The CompCor method addresses confounding effects without affecting intrinsic functional connectivity (Chai et al., 2012) while improving specificity, sensitivity, and validity of subsequent functional connectivity analyses such as false positive anticorrelated activity (Whitfield‐Gabrieli & Nieto‐Castanon, 2012). Data were not band‐pass filtered or detrended since these processes underestimate the effect of non‐BOLD fluctuations, remove BOLD‐related fluctuations, and discard low‐frequency components (Evans et al., 2015; Kundu et al., 2012) that mediate rsFC and are necessary for detecting functionally relevant networks (Biswal et al., 1995; Fransson, 2005; M. D. Greicius et al., 2003).

#### 2.6.2 Functional connectivity

We used exploratory whole‐brain seed‐based analyses to determine the effects of the two rTMS protocols on rsFC using the CONN toolbox. Temporal correlations of BOLD signal during rest were computed between a seed region‐of‐interest (ROI) from which reference time series were extracted and all other voxels in the brain, thus yielding seed ROI‐specific spatial functional maps (seed‐to‐voxel analysis; Biswal et al., 1995). Two seed ROIs were explored: the stimulation site at the visual cortex (V1) associated with the visual network and the posterior cingulate cortex/precuneus associated with the DMN. For V1, a 10 mm radius sphere ROI was created externally in FSLeyes (FMRIB, Oxford, UK; www.fmrib.ox.ac.uk/fsl). The V1 seed ROIs were created for each participant individually, centered at the stimulation site and positioned closest to the posterior surface of the occipital pole (Brodmann area [BA] 17), which received the strongest stimulation since it was the region closest to the coil (average MNI coordinates: *x* = 1, *y* = −81, *z* = 15; see Table S1 for individual MNI coordinates). The V1 seed ROI corresponded with both the Brainsight target stimulation site and the MRS volume‐of‐interest. The DMN seed ROI was selected from the CONN DMN atlas. For the DMN, a 10 mm radius sphere ROI was placed in the posterior cingulate cortex/precuneus (BA 23/31; MNI coordinates: *x* = −5, *y* = −52, *z* = 40) in all participants. Overlapping posterior cingulate cortex/precuneus regions are considered a critical node in the DMN (Damoiseaux et al., 2006; Grecius et al., 2003; Gusnard et al., 2001) and are shown to extract reliable patterns of DMN functional connectivity using seed‐based analyses (e.g., Fox et al., 2005; Fransson, 2005; M. D. Greicius et al., 2003).

First‐level analyses correlated the average BOLD time course between each seed ROI to whole‐brain voxels (one dimension, no temporal expansion of derivatives, no frequency decomposition) to create rsFC maps for each visit and participant independently. A weighted general linear model and bivariate Pearson's correlations were used (with no hemodynamic response function weighting). The correlation coefficients represent the level of association between two time series that reflect the relative degree of functional connectivity of each seed and each voxel in the brain. The resulting weighted correlation coefficients were converted to normally distributed *z*‐scores using Fisher's transformation.

First‐level individual rsFC maps were subsequently used for second‐level general linear model analyses to investigate significant changes in seed‐to‐voxel rsFC between pre‐rTMS and follow‐up visits for each rTMS group and seed ROI separately. Paired *t*‐tests with a covariate were calculated to investigate whole‐brain differences in rsFC between pre‐rTMS and each follow‐up visit. rTMS groups were analyzed independently due to different follow‐up intervals. The number of degrees of freedom from ME denoised data for each participant and visit were entered as a covariate of no interest for subject‐ and group‐level analyses to control for and avoid inflated test statistics and false positive results (Kundu et al., 2017). Nonparametric statistics (1000 permutations; Pernet et al., 2015) were chosen to control for false positive rates and to provide added protection against potential violations of parametric assumptions with small sample sizes (Eklund et al., 2016). Significant clusters were investigated with post hoc simple effects analyses to identify the direction of rsFC effects (i.e., increase or decrease) between the pre‐rTMS visit and each post‐rTMS follow‐up visit. The resulting rsFC maps were thresholded at a whole‐brain uncorrected voxel‐level (height) threshold of *p* < .001, cluster‐mass (extent) threshold of *p* < .05 with false discovery rate (FDR) correction for multiple comparisons, and a minimum cluster size of 25 voxels. These a priori thresholds were chosen in accordance with CONN toolbox guidelines for supporting strong focal effects as opposed to weaker diffuse effects (uncorrected voxel‐level threshold of *p* < .01), and to further constrain false positive effects. Positive and negative correlations (two‐sided) were examined. Regions showing significant changes in rsFC associated with the seed ROIs between pre‐rTMS and follow‐up visits were identified using the following CONN implemented atlases: Harvard‐Oxford cortical and subcortical probabilistic (25% probability) structural atlases (Desikan et al., 2006), the Automated Anatomical Labeling atlas to parcellate cerebellar areas (Tzourio‐Mazoyer et al., 2002), and the BA atlas (Brodmann, 1909, 1910).

To investigate how rsFC within these networks are associated with GABA+ and Glx concentrations following the two rTMS protocols, we repeated the above procedure with multiple regression analyses. The metabolite concentrations obtained from our MRS study were acquired from the same V1 stimulation site seed ROI used for rsFC and were included as a covariate of interest in second‐level analyses. We still controlled for the number of degrees of freedom from ME denoised data (covariate of no interest). These analyses evaluated the correlation between change in rsFC (difference in connectivity pre‐ and post‐rTMS) and change in metabolite concentration (difference in GABA+/Glx concentration pre‐ and post‐rTMS) in the networks of interest. Analyses were performed to compare pre‐ and post‐rTMS for each metabolite, seed ROI, and rTMS group separately.

## 3 RESULTS

### 3.1 Effect of low‐frequency rTMS on functional connectivity

**FIGURE 3:**
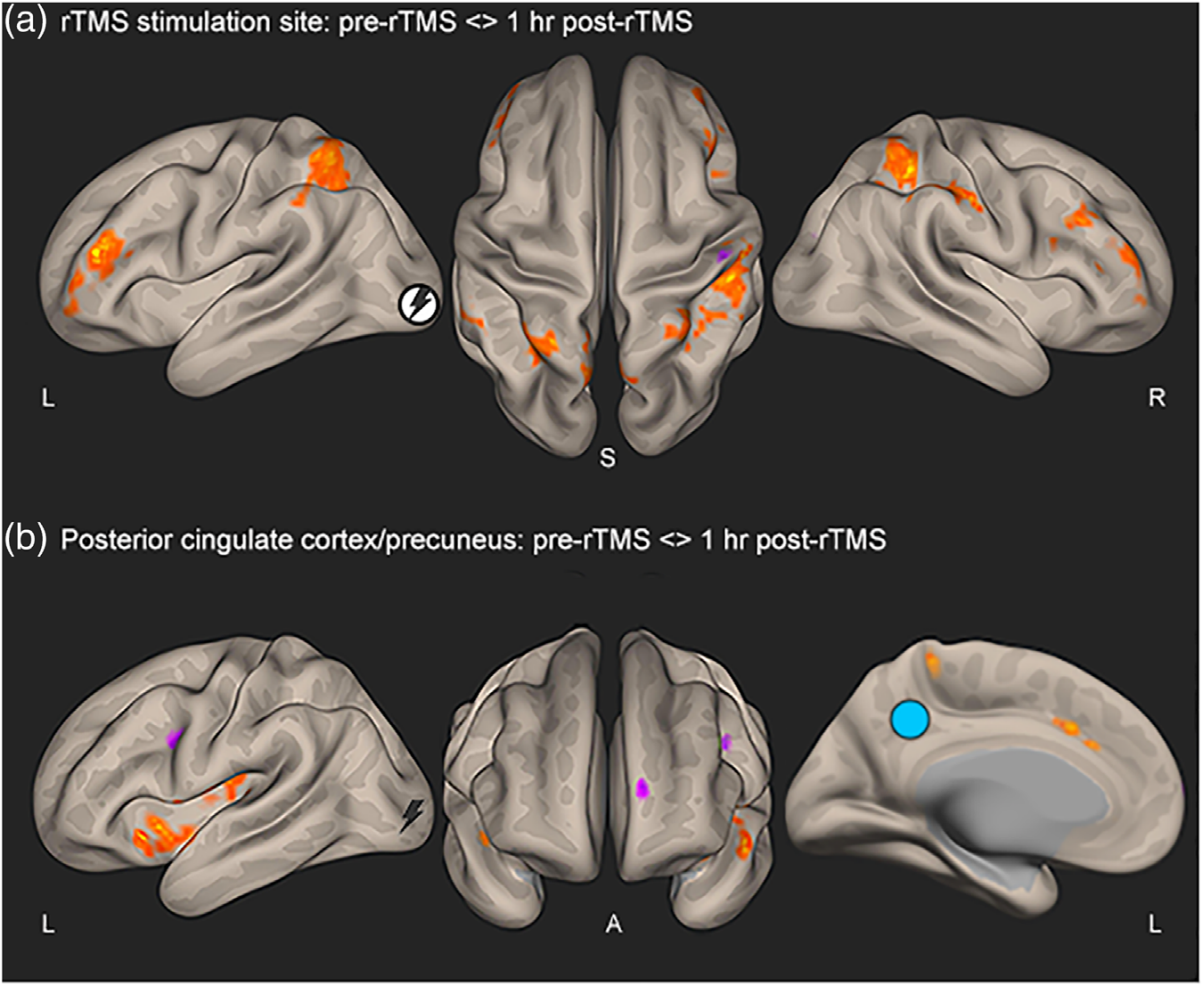
Resting‐state maps of change in functional connectivity following a single rTMS session to the visual cortex. (a) Regions showing a significant change in connectivity with the stimulation site seed (white circle). (b) Regions showing a significant change in connectivity with the posterior cingulate cortex/precuneus seed (light blue circle). Images show a *p*‐value map (*p* < .001). Orange/yellow regions show a positive change in correlation with the seed (decrease in rsFC at 1 h post‐rTMS), while pink/purple regions show a negative change in correlation with the seed (increase in rsFC at 1 h post‐rTMS). Lightning bolt shows the stimulation site at the visual cortex (V1). Abbreviations: A, anterior; I, inferior; L, left hemisphere; R, right hemisphere; S, superior; rTMS, repetitive transcranial magnetic stimulation; rsFC, resting‐state functional connectivity

**FIGURE 4:**
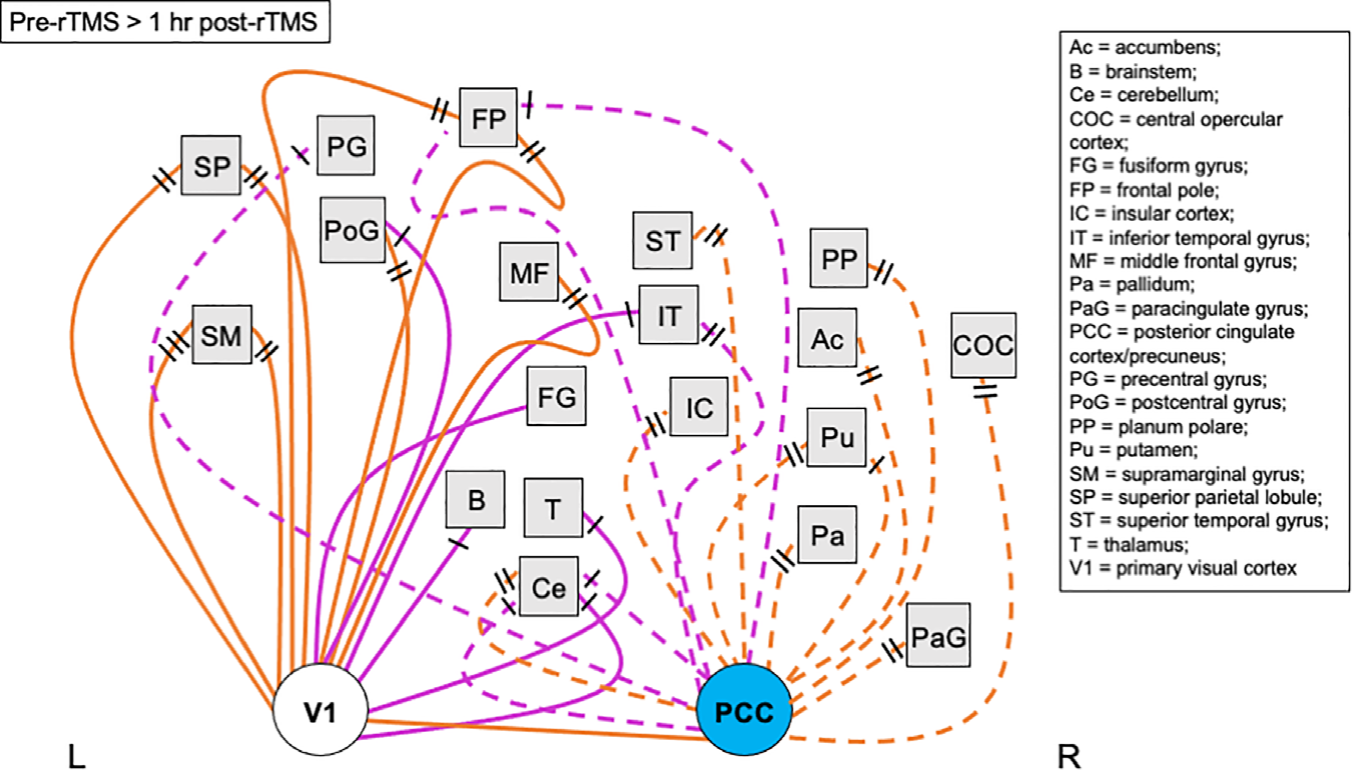
Summary of functional connectivity changes following a single rTMS session to the visual cortex. Image shows a visual summary of rsFC changes presented in Figure 3 and Tables 1 and 2. Significant changes in rsFC were observed only at 1 h post‐rTMS compared with pre‐rTMS (baseline) following a single rTMS session to the visual cortex (no significant changes observed immediately post‐rTMS). Nodes/regions (squares) showing a significant change in connectivity with the stimulation site seed (V1, white circle) are mapped using a solid line (direct stimulation effect). Regions showing a significant change in connectivity with the posterior cingulate cortex/precuneus seed (PCC, light blue circle) are shown with a dashed line (indirect stimulation effect). Orange lines show a positive change in correlation with the seed (decrease in rsFC at 1 h post‐rTMS; positive effect size), while pink lines show a negative change in correlation with the seed (increase in rsFC at 1 h post‐rTMS; negative effect size). Black dashes represent the direction of correlations: no black lines = correlated, one black line = anticorrelated pre‐rTMS, two black lines = anticorrelated post‐rTMS, three black lines = anticorrelated pre‐ and post‐rTMS. Lines connecting to nodes on the left of the square represent changes to that region in the left hemisphere, whereas lines connecting nodes to the right of the square represent changes to that region in the right hemisphere. Nodes positioned in the midline are connected with lines to the bottom edge of the square. There is no hemisphere differentiation or otherwise for the seed points. Image is not anatomically correct and does not distinguish between further subregions/locations within the node (unlike the detailed tables). Abbreviations: L, left hemisphere; R, right hemisphere; rTMS, repetitive transcranial magnetic stimulation; rsFC, resting‐state functional connectivity

Regions demonstrating significant changes in rsFC with the stimulation site (V1) and posterior cingulate cortex/precuneus seed ROIs following a single rTMS session are provided in Tables 1 and 2, respectively. For both seed ROIs, there were no significant changes in rsFC with correlated regions immediately following cessation of rTMS compared with pre‐rTMS. However, significant changes in rsFC were detected 1 h after rTMS had ceased. The stimulation site showed significant changes in rsFC with frontal, parietal, temporal, and occipital lobe regions, as well as the brainstem, thalamus, and cerebellum (Table 1; Figure 3a). The posterior cingulate cortex/precuneus showed significant changes in rsFC with frontal and temporal lobe regions, cingulate gyrus, cerebellum, and basal ganglia (putamen, pallidus, accumbens) (Table 2; Figure 3b). A summary of effects is provided in Figure 4.

**TABLE 1:** Regions showing altered functional connectivity with the visual cortex (stimulation site) following a single rTMS session

		MNI coordinates				
Contrast/region	BA	*x*	*y*	*z*	Voxels	Effect size
Pre‐rTMS *>* immediate post‐rTMS						
N.S.						
Pre‐rTMS > 1 h post‐rTMS						
R superior parietal lobule	7	29	−46	50	628	0.28^b^
L superior parietal lobule	7	−34	−60	53	465	0.25^b^
R anterior supramarginal gyrus	40	48	−30	39	183	0.25^b^
R posterior supramarginal gyrus	40	59	−46	45	36	0.17^b^
L posterior supramarginal gyrus	40	−53	−49	15	54	0.14^a^, ^b^
R postcentral gyrus	2	51	−19	37	105	0.19^b^
R postcentral gyrus	2	51	−14	50	99	−0.18^a^
Precuneus	7	2	−66	48	489	0.23
R middle frontal gyrus	9	48	16	31	389	0.24^b^
R frontal Pole	46	51	43	18	239	0.29^b^
L frontal Pole	46	−44	38	12	303	0.23^b^
Brainstem		−9	−33	−10	129	−0.23^a^
L posterior inferior temporal gyrus	20	−50	−19	−29	111	−0.13^a^
R thalamus		7	−27	15	81	−0.17^a^
R cerebellum crus 1		40	−76	−23	66	−0.22^a^
L fusiform gyrus	19	−31	−74	−7	54	−0.18

*Notes*: The columns list (from left to right) regions showing significant differences in rsFC with the stimulation site between pre‐ and post‐rTMS visits (uncorrected *p *< .001; cluster‐mass *p *< .05 FDR corrected), the associated BA, peak MNI coordinates of the cluster, cluster voxel size (≥25 voxels), and effect size. Effect sizes represent the average difference in Fisher‐transformed correlation coefficients between visits (pre‐TMS visit minus the post‐rTMS visit) for the stimulation site (seed) and the correlated region. A positive effect size indicates a decrease in rsFC at the post‐rTMS visit, while a negative effect size indicates an increase in rsFC at the post‐rTMS visit.

Abbreviations: BA, Brodmann area; FDR, false discovery rate; L, left hemisphere; MNI, Montreal Neurological Institute; N.S., no significant difference; rTMS, repetitive transcranial magnetic stimulation; rsFC, resting‐state functional connectivity; R, right hemisphere.

^a^ Anticorrelated pre‐rTMS.

^b^ Anticorrelated post‐rTMS.

**TABLE 2:** Regions showing altered functional connectivity with the posterior cingulate cortex/precuneus following a single rTMS session to the visual cortex

		MNI coordinates				
Contrast/region	BA	*x*	*y*	*z*	Voxels	Effect size
Pre‐rTMS > immediate post‐rTMS						
N.S.						
Pre‐rTMS > 1 h post‐rTMS						
L insular cortex	13	−42	11	−4	633	0.26^b^
R frontal pole	10	−1	65	7	34	−0.22^a^
L frontal pole	10	−14	65	9	254	−0.25
R cerebellum crus 2		37	−74	−45	54	−0.27^a^
L cerebellum crus 2		−42	−63	−42	36	−0.22^a^
L cerebellum 8		−14	−68	−37	39	0.12^b^
L cerebellum 7b		−6	−79	−40	57	0.17^b^
L cerebellum 6		−28	−49	−37	81	0.19^b^
R putamen		24	13	−10	117	0.19^a^
L putamen		−34	−6	1	156	0.17^b^
L pallidum		−20	0	−7	81	0.24^b^
R accumbens		10	11	−10	45	0.20^b^
R posterior superior temporal gyrus	22	51	−11	−10	135	0.18^b^
R inferior temporal gyrus	20	65	−22	−29	30	−0.20^a^
L planum polare	13	−47	−8	−1	60	0.31^b^
L precentral gyrus	6	−39	0	26	120	−0.20^a^
L paracingulate gyrus	32	−12	16	34	102	0.28^b^
L central opercular cortex	13	−42	−8	12	90	0.17^b^

*Notes*: The columns list (from left to right) regions showing significant differences in rsFC with the posterior cingulate cortex/precuneus between pre‐ and post‐rTMS visits (uncorrected *p *< .001; cluster‐mass *p *< .05 FDR corrected), the associated BA, peak MNI coordinates of the cluster, cluster voxel size (≥25 voxels), and effect size. Effect sizes represent the average difference in Fisher‐transformed correlation coefficients between visits (pre‐TMS visit minus the post‐rTMS visit) for the posterior cingulate cortex/precuneus (seed) and the correlated region. A positive effect size indicates a decrease in rsFC at the post‐rTMS visit, while a negative effect size indicates an increase in rsFC at the post‐rTMS visit.

Abbreviations: BA, Brodmann area; FDR, false discovery rate; L, left hemisphere; MNI, Montreal Neurological Institute; N.S., no significant difference; rTMS, repetitive transcranial magnetic stimulation; rsFC, resting‐state functional connectivity; R, right hemisphere.

^a^ Anticorrelated pre‐rTMS.

^b^ Anticorrelated post‐rTMS.

There were no significant changes in rsFC between the stimulation site and correlated regions immediately following accelerated rTMS. Only the middle temporo‐occipital region showed a significant decrease in rsFC with the posterior cingulate cortex/precuneus at 1 week post‐rTMS compared with pre‐rTMS (BA 22; MNI coordinates: *x* = 54, *y* = −41, *z* = 1; voxels = 411 mm^3^; effect size = 0.21). However, using a less conservative voxel‐level threshold of *p* < .01 revealed diffuse weak changes in rsFC between the stimulation site and correlated regions only at 24 h post‐rTMS (see Table S2), and at 1 week post‐rTMS for regions correlated with the posterior cingulate cortex/precuneus (see Table S3).

The average difference in connectivity strength between pre‐ and post‐rTMS visits across participants for each seed ROI and correlated regions are represented by effect size values in Tables 1 and 2 and Tables S2 and S3 in.

### 3.2 Relationship between metabolites and functional connectivity using low‐frequency rTMS

Multiple regression analyses demonstrated a significant correlation between pre‐ and post‐rTMS differences in rsFC and metabolite concentrations in networks associated with our seed ROIs. These findings suggest that changes in rsFC are related to GABA+ and/or Glx changes following 1 Hz rTMS to the visual cortex. Metabolite concentrations are originally reported in Rafique and Steeves (2020). For the single rTMS group, mean_concentration_ ± SEM (in institutional units [i.u.]) for metabolites were as follows: GABA+ pre‐rTMS (baseline) = 3.67 ± 0.26, immediate post‐rTMS = 3.92 ± 0.31, 1 h post‐rTMS = 3.73 ± 0.3; Glx pre‐rTMS (baseline) = 7.87 ± 0.45, immediate post‐rTMS = 8.0 ± 0.48, 1 h post‐rTMS = 7.84 ± 0.48. For the accelerated rTMS group, mean_concentration_ ± SEM (i.u.): GABA+ pre‐rTMS (baseline) = 3.6 ± 0.11, immediate post‐rTMS = 3.32 ± 0.11, 24 h post‐rTMS = 3.38 ± 0.11, 1 week post‐rTMS = 3.78 ± 0.18; Glx pre‐rTMS (baseline) = 7.43 ± 0.19, immediate post‐rTMS = 7.13 ± 0.28, 24 h post‐rTMS = 7.31 ± 0.31, 1 week post‐rTMS = 7.6 ± 0.32.

**FIGURE 5:**
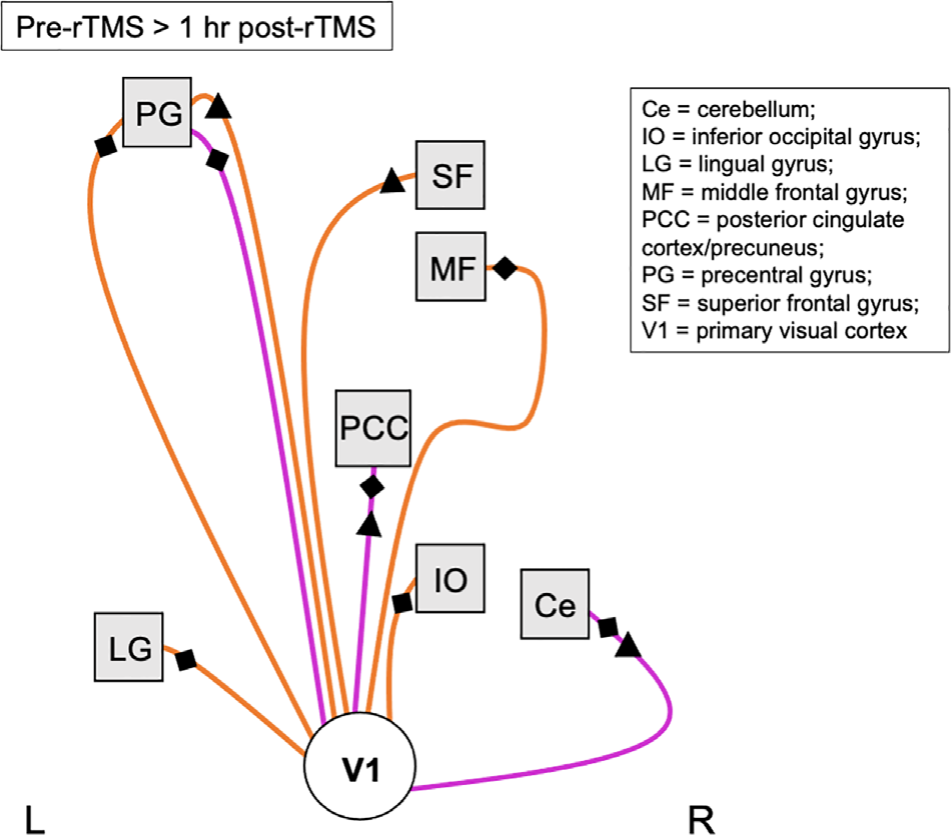
Summary of altered functional connectivity with the visual cortex (stimulation site) related to GABA+ and Glx changes following a single rTMS session. Image shows a visual summary of rsFC changes associated with changes in GABA+ (diamonds) and Glx (triangles) concentrations that are presented in Table 3. Significant changes in rsFC were only observed at 1 h post‐rTMS compared with pre‐rTMS (baseline) following a single rTMS session to the visual cortex (no significant changes observed immediately post‐rTMS). Nodes/regions (squares) showing a significant change in connectivity with the stimulation site seed (V1, white circle) are mapped using a solid line to indicate a direct stimulation effect. Orange lines show a positive change in correlation with the seed (decrease in rsFC at 1 h post‐rTMS; positive effect size), while pink lines show a negative change in correlation with the seed (increase in rsFC at 1 h post‐rTMS; negative effect size). Lines connecting to nodes on the left of the square represent changes to that region in the left hemisphere, whereas lines connecting nodes to the right of the square represent changes to that region in the right hemisphere. Nodes positioned in the midline are connected with lines to the bottom edge of the square. There is no hemisphere differentiation or otherwise for the seed points. Image is not anatomically correct and does not distinguish between further subregions/locations within the node (unlike the detailed tables). Abbreviations: L, left hemisphere; R, right hemisphere; rTMS, repetitive transcranial magnetic stimulation; rsFC, resting‐state functional connectivity

**FIGURE 6:**
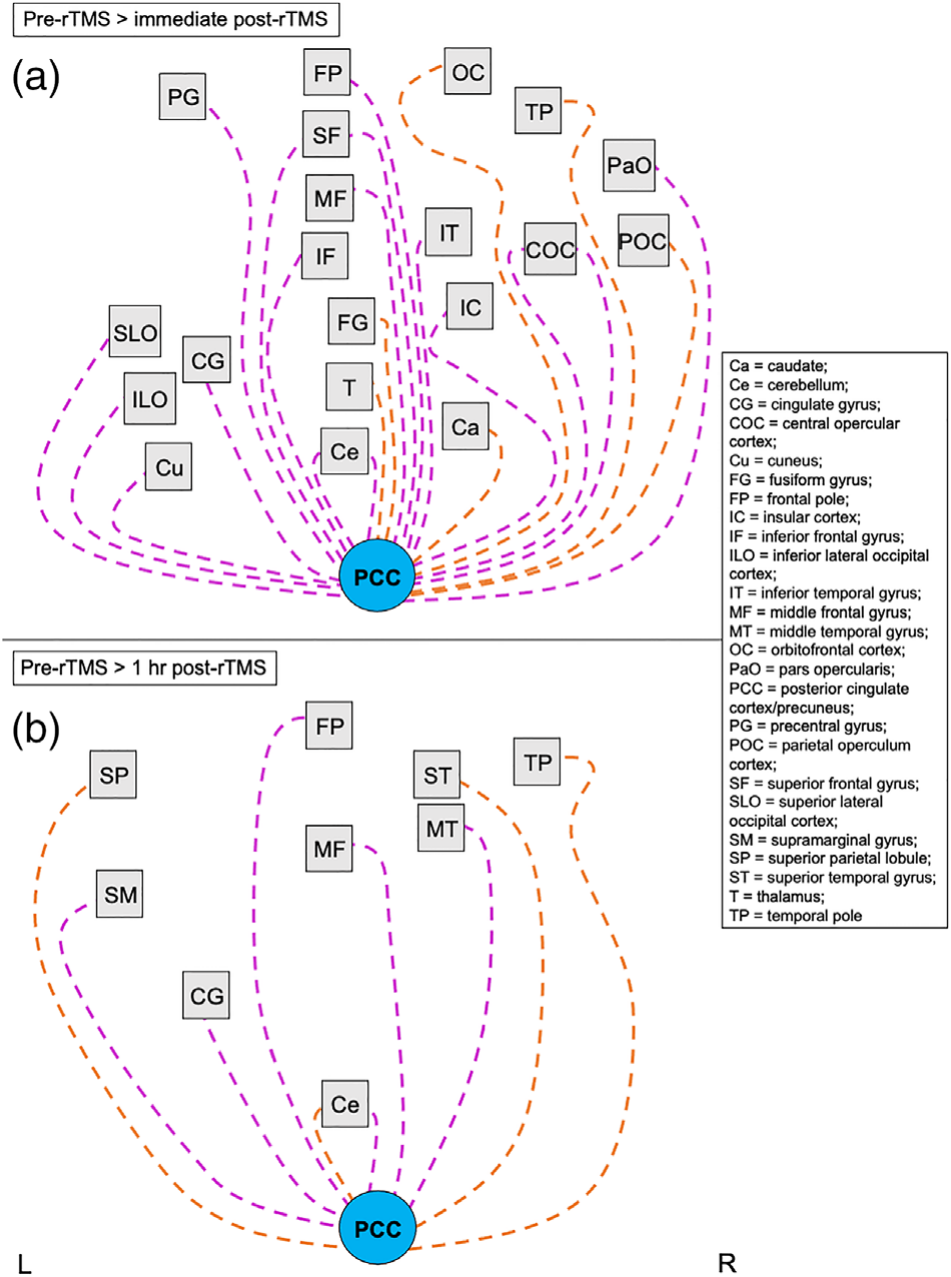
Summary of altered functional connectivity with the posterior cingulate cortex/precuneus related to GABA+ changes following a single rTMS session to the visual cortex. Images show a visual summary of rsFC changes associated with changes in GABA+ concentrations that are presented in Table 4 (no significant differences were associated with Glx concentrations). Significant changes in rsFC were observed at (a) immediate post‐rTMS and (b) 1 h post‐rTMS compared with pre‐rTMS (baseline) following a single rTMS session to the visual cortex. Nodes/regions (squares) showing a significant change in connectivity with the posterior cingulate cortex/precuneus seed (PCC, light blue circle) are shown with a dashed line to indicate an indirect stimulation effect. Orange lines show a positive change in correlation with the seed (decrease in rsFC at 1 h post‐rTMS; positive effect size), while pink lines show a negative change in correlation with the seed (increase in rsFC at 1 h post‐rTMS; negative effect size). Lines connecting to nodes on the left of the square represent changes to that region in the left hemisphere, whereas lines connecting nodes to the right of the square represent changes to that region in the right hemisphere. Nodes positioned in the midline are connected with lines to the bottom edge of the square. There is no hemisphere differentiation or otherwise for the seed points. Images are not anatomically correct and do not distinguish between further subregions/locations within the node (unlike the detailed tables). Abbreviations: L, left hemisphere; R, right hemisphere; rTMS, repetitive transcranial magnetic stimulation; rsFC, resting‐state functional connectivity

Significant changes in rsFC following a single rTMS session for both seed ROIs, the stimulation site and posterior cingulate cortex/precuneus, are provided in Tables 3 and 4, respectively. Significant changes in rsFC between the stimulation site (V1) and correlated regions associated with GABA+/Glx were only apparent 1 h after rTMS ceased. The stimulation site showed significant changes in rsFC with frontal, parietal, and occipital lobe regions, and the cerebellum (Table 3; for a summary of effects, see Figure 5). However, in the case of the posterior cingulate cortex/precuneus seed, significant changes in rsFC with correlated regions were only associated with GABA+ and not Glx. Effects with the posterior cingulate cortex/precuneus were detected immediately after rTMS ceased and continued until 1 h post‐rTMS. The posterior cingulate cortex/precuneus showed significant changes in rsFC with frontal, parietal, occipital, and temporal lobe regions, as well as the cingulate gyrus, basal ganglia (caudate), thalamus, and cerebellum (Table 4; for a summary of effects, see Figure 6). Notably, effects associated with the posterior cingulate cortex/precuneus were more widespread and unstable, showing variable changes in network connectivity at 1 h compared with immediately post‐rTMS.

**TABLE 3:** Regions showing altered functional connectivity with the visual cortex (stimulation site) related to GABA+ and Glx changes following a single rTMS session

		MNI coordinates				
Metabolite/contrast/region	BA	*x*	*y*	*z*	Voxels	Effect size
GABA+						
Pre‐rTMS > immediate post‐rTMS						
N.S.						
Pre‐rTMS > 1 h post‐rTMS						
R precentral gyrus	4	21	−14	50	147	−0.74
L precentral gyrus	6	−50	−3	37	36	0.74
R lingual gyrus	17	18	−96	4	117	1.02
L inferior occipital gyrus	18	−30	−99	−3	112	1.21
Precuneus		21	−60	26	45	−0.74
R middle frontal gyrus	6	45	13	48	45	0.62
R cerebellum 9		13	−46	−59	68	−0.68
R cerebellum 7b		29	−76	−53	48	−0.58
Glx						
Pre‐rTMS > immediate post‐rTMS						
N.S.						
Pre‐rTMS > 1 h post‐rTMS						
L superior frontal gyrus	6	−12	41	53	102	0.28
R cerebellum 8		18	−44	−56	72	−0.21
Precuneus	7	10	−71	48	45	−0.23
R precentral gyrus		26	−17	75	42	0.26

*Notes*: The columns list (from left to right) regions showing significant differences in rsFC with the stimulation site that are associated with GABA+/Glx concentrations between pre‐ and post‐rTMS visits (uncorrected *p *< .001; cluster‐mass *p *< .05 FDR corrected), the associated BA, peak MNI coordinates of the cluster, cluster voxel size (≥25 voxels), and effect size. Effect sizes represent Fisher‐transformed regression coefficients as a ratio of change in rsFC between the stimulation site (seed) and the correlated region per unit change in metabolite concentration. A positive effect size indicates a decrease in rsFC at the post‐rTMS visit, while a negative effect size indicates an increase in rsFC at the post‐rTMS visit. Average change in GABA+ concentration between pre‐rTMS and immediate post‐rTMS = −0.176 i.u., and pre‐rTMS and 1 h post‐rTMS = −0.057 i.u. Average change in Glx concentration between pre‐rTMS and immediate post‐rTMS = −0.039 i.u., and pre‐rTMS and 1 h post‐rTMS = 0.109 i.u.

Abbreviations: BA, Brodmann area; FDR, false discovery rate; i.u., institutional units; Glx, glutamate + glutamine; GABA+, GABA + macromolecules; L, left hemisphere; MNI, Montreal Neurological Institute; N.S., no significant difference; R, right hemisphere; rsFC, resting‐state functional connectivity; rTMS, repetitive transcranial magnetic stimulation.

**TABLE 4:** Regions showing altered functional connectivity with the posterior cingulate cortex/precuneus related to GABA+ changes following a single rTMS session to the visual cortex

		MNI coordinates				
Contrast/region	BA	*x*	*y*	*z*	Voxels	Effect size
Pre‐rTMS > immediate post‐rTMS						
R superior frontal gyrus	6	18	3	69	126	−0.31
L superior frontal gyrus	6	−1	8	67	374	−0.38
L inferior frontal gyrus	45	−47	22	7	93	−0.30
R middle frontal gyrus	8	29	24	34	72	−0.34
R frontal pole	68	35	38	45	68	−0.28
L orbitofrontal cortex	47	−20	19	−26	27	0.10
R caudate		15	16	15	207	0.32
R precentral gyrus	6	45	5	34	135	−0.45
R thalamus		18	−25	12	81	0.23
L cerebellum 9		−20	−52	−45	81	−0.22
L cerebellum 8		−20	−66	−42	36	−0.12
L cerebellum 6		−31	−63	−26	27	−0.21
R cerebellum crus 2		5	−82	−26	45	−0.24
R cerebellum crus 1		56	−46	−31	50	−0.21
L cerebellum crus 1		−39	−82	−29	27	−0.23
L superior lateral occipital cortex		−44	−71	23	63	−0.47
L inferior lateral occipital cortex		−47	−66	−7	27	−0.16
Anterior cingulate gyrus	24	−6	3	29	50	−0.25
R temporal pole		21	8	−48	41	0.23
L inferior temporal gyrus	37	−47	−55	−15	27	−0.11
R pars opercularis	44/45	56	13	4	90	−0.38
R parietal operculum cortex	13	59	−30	23	117	0.30
R central opercular cortex	13	43	8	4	30	−0.26
L central opercular cortex	43	−58	−6	7	36	−0.34
L cuneus	18	−4	−98	15	30	−0.24
L insular Cortex	13	−42	0	−4	27	−0.22
R fusiform gyrus	19	26	−79	−15	27	0.09
Pre‐rTMS > 1 h post‐rTMS						
R temporal pole	22	56	11	10	229	0.84
R cerebellum crus 2		51	−63	−45	68	−0.86
R cerebellum 8		29	−57	−59	30	−0.60
L cerebellum 8		−20	−52	−59	219	0.84
L frontal pole	10	−6	68	−1	99	−0.96
R middle frontal gyrus	8	35	27	34	36	−1.25
R anterior superior temporal gyrus	22	65	−6	−1	98	1.21
R posterior middle temporal gyrus		59	−14	−23	54	−0.61
L posterior supramarginal gyrus	40	−50	−46	42	63	−0.83
L superior parietal lobule	7	−34	−44	67	39	0.83
Posterior cingulate gyrus	31	−4	−33	39	45	−0.77

*Notes*: The columns list (from left to right) regions showing significant differences in rsFC with the posterior cingulate cortex/precuneus that are associated with GABA+ concentrations between pre‐ and post‐rTMS visits (uncorrected *p *< .001; cluster‐mass *p *< .05 FDR corrected), the associated BA, peak MNI coordinates of the cluster, cluster voxel size (≥25 voxels), and effect size. Effect sizes represent Fisher‐transformed regression coefficients as a ratio of change in rsFC between the posterior cingulate cortex/precuneus (seed) and the correlated region per unit change in GABA+ concentration. A positive effect size indicates a decrease in rsFC at the post‐rTMS visit, while a negative effect size indicates an increase in rsFC at the post‐rTMS visit. Average change in GABA+ concentration between pre‐rTMS and immediate post‐rTMS = −0.176 i.u., and pre‐rTMS and 1 h post‐rTMS = −0.057 i.u. There were no significant differences in rsFC in regions associated with Glx concentrations. Abbreviations: BA, Brodmann area; FDR, false discovery rate; i.u., institutional units; Glx, glutamate + glutamine; GABA+, GABA + macromolecules; L, left hemisphere; MNI, Montreal Neurological Institute; N.S., no significant difference; R, right hemisphere; rsFC, resting‐state functional connectivity; rTMS, repetitive transcranial magnetic stimulation.

**FIGURE 7:**
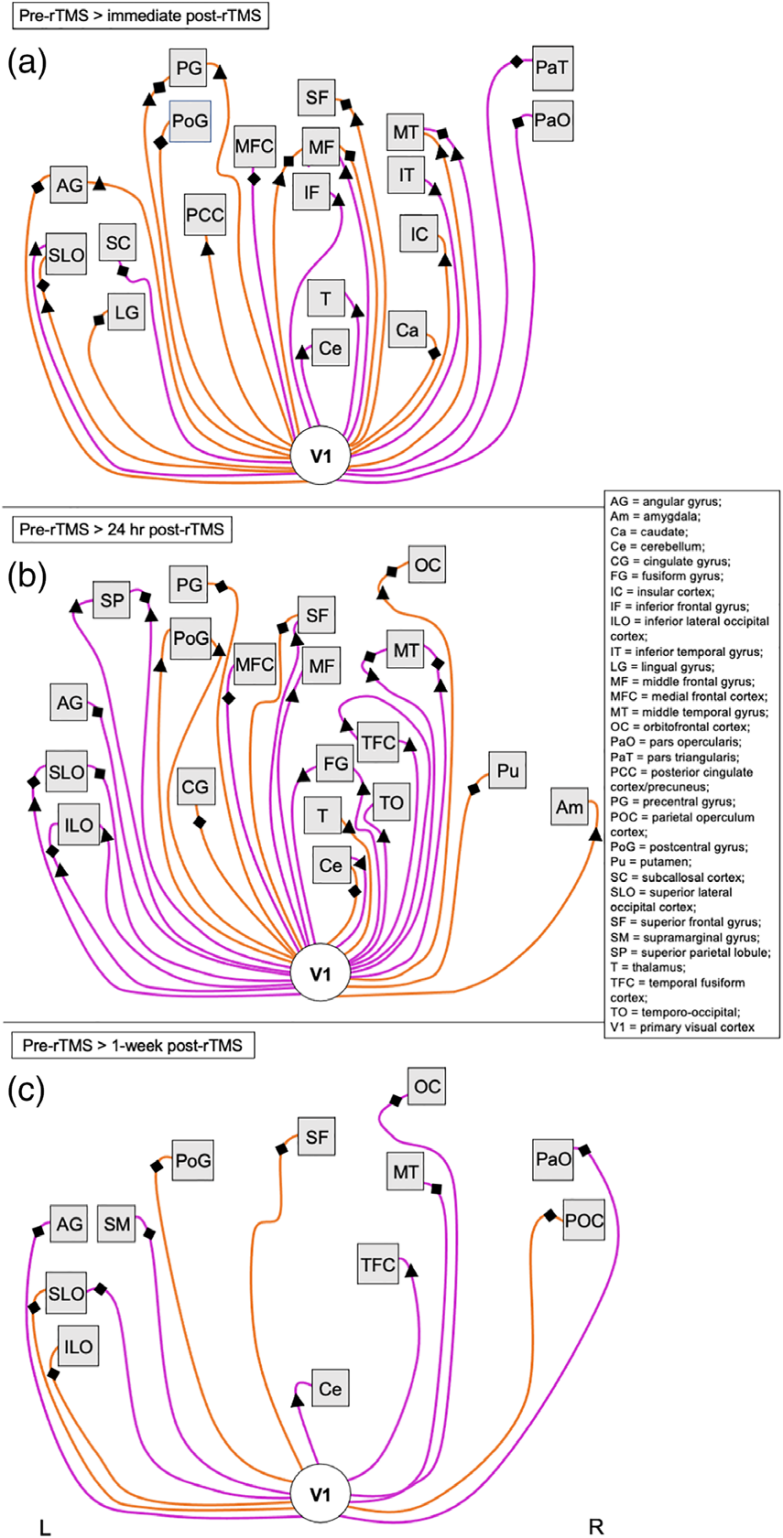
Summary of altered functional connectivity with the visual cortex (stimulation site) related to GABA+ and Glx changes following accelerated rTMS sessions. Images show a visual summary of rsFC changes associated with changes in GABA+ (diamonds) and Glx (triangles) concentrations that are presented in Table 5. Significant changes in rsFC were observed at (a) immediate post‐rTMS, (b) 24 h post‐rTMS, and (c) 1 week post‐rTMS compared with pre‐rTMS (baseline) following accelerated rTMS sessions to the visual cortex. Nodes/regions (squares) showing a significant change in connectivity with the stimulation site seed (V1, white circle) are mapped using a solid line to indicate a direct stimulation effect. Orange lines show a positive change in correlation with the seed (decrease in rsFC at 1 h post‐rTMS; positive effect size), while pink lines show a negative change in correlation with the seed (increase in rsFC at 1 h post‐rTMS; negative effect size). Lines connecting to nodes on the left of the square represent changes to that region in the left hemisphere, whereas lines connecting nodes to the right of the square represent changes to that region in the right hemisphere. Nodes positioned in the midline are connected with lines to the bottom edge of the square. There is no hemisphere differentiation or otherwise for the seed points. Images are not anatomically correct and do not distinguish between further subregions/locations within the node (unlike the detailed tables). Abbreviations: L, left hemisphere; R, right hemisphere; rTMS, repetitive transcranial magnetic stimulation; rsFC, resting‐state functional connectivity

**FIGURE 8:**
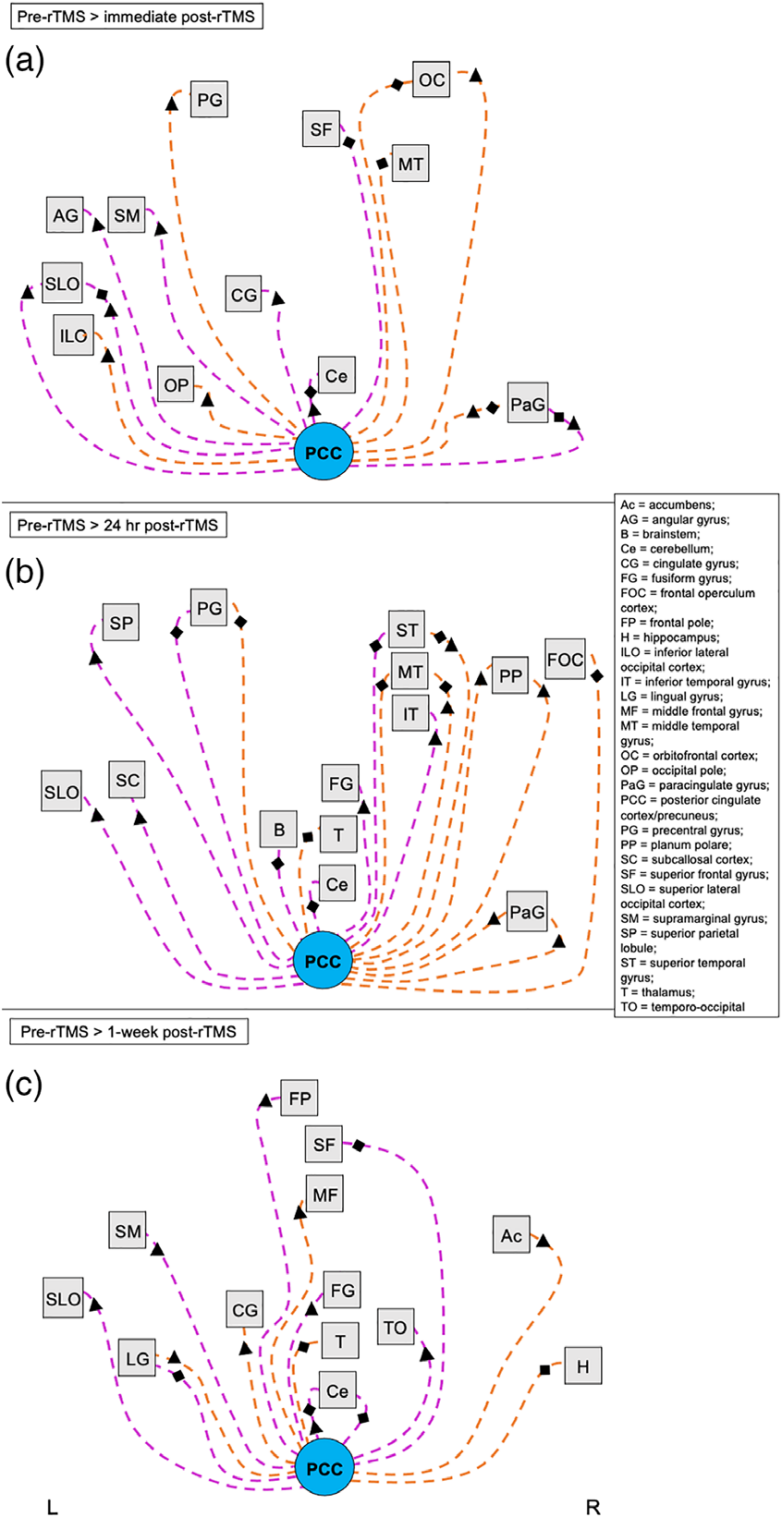
Summary of altered functional connectivity with the posterior cingulate cortex/precuneus related to GABA+ and Glx changes following accelerated rTMS sessions to the visual cortex. Images show a visual summary of rsFC changes associated with changes in GABA+ (diamonds) and Glx (triangles) concentrations that are presented in Table 6. Significant changes in rsFC were observed at (a) immediate post‐rTMS, (b) 24 h post‐rTMS, and (c) 1 week post‐rTMS compared with pre‐rTMS (baseline) following accelerated rTMS sessions to the visual cortex. Nodes/regions (squares) showing a significant change in connectivity with the posterior cingulate cortex/precuneus seed (PCC, light blue circle) are shown with a dashed line to indicate an indirect stimulation effect. Orange lines show a positive change in correlation with the seed (decrease in rsFC at 1 h post‐rTMS; positive effect size), while pink lines show a negative change in correlation with the seed (increase in rsFC at 1 h post‐rTMS; negative effect size). Lines connecting to nodes on the left of the square represent changes to that region in the left hemisphere, whereas lines connecting nodes to the right of the square represent changes to that region in the right hemisphere. Nodes positioned in the midline are connected with lines to the bottom edge of the square. There is no hemisphere differentiation or otherwise for the seed points. Images are not anatomically correct and do not distinguish between further subregions/locations within the node (unlike the detailed tables). Abbreviations: L, left hemisphere; R, right hemisphere; rTMS, repetitive transcranial magnetic stimulation; rsFC, resting‐state functional connectivity

Tables 5 and 6 show significant changes in rsFC following the accelerated rTMS protocol for the stimulation site and posterior cingulate cortex/precuneus seed ROIs, respectively. A summary of effects is provided in Figures 7 and 8, respectively. With accelerated rTMS, significant changes in rsFC between the seed ROIs and correlated regions associated with GABA+ and Glx were apparent immediately after rTMS, and changes continued to 1 week post‐rTMS. Widespread changes in rsFC were observed to the frontal, parietal, occipital, and temporal lobes, cingulate gyrus, basal ganglia, thalamus, amygdala, brainstem, and cerebellum. These effects were unstable and continued to change even at 1 week post‐rTMS as observed from the inconsistent involvement of interconnected regions and/or connectivity changes at the different follow‐visits. For example, the left superior lateral occipital cortex shows a decrease in rsFC with the stimulation site that is associated with a change in GABA+ immediately post‐rTMS, while an increase in rsFC is observed between these regions at 24 h post‐rTMS (Table 5).

**TABLE 5:** Regions showing altered functional connectivity with the visual cortex (stimulation site) related to GABA+ and Glx changes following accelerated rTMS sessions

		MNI coordinates				
Metabolite/contrast/region	BA	*x*	*y*	*z*	Voxels	Effect size
GABA+						
Pre‐rTMS > immediate post‐rTMS						
R superior frontal gyrus	8	24	38	48	486	0.63
L pars opercularis	44	−55	13	15	273	−0.63
L pars triangularis	45	−50	27	4	27	−0.60
R middle frontal gyrus	9	45	24	34	99	0.53
L middle frontal gyrus	6	−34	5	59	81	0.57
Medial frontal cortex	11	−1	41	−37	50	−0.45
R middle temporal gyrus	21/22	56	−22	−7	72	−0.59
L lingual gyrus	19	−25	−74	4	141	0.52
L superior lateral occipital cortex	7	−31	−63	50	27	0.56
L precentral gyrus	6	−53	0	39	81	0.63
L postcentral gyrus	40	−53	−30	53	27	0.32
L angular gyrus	39	−42	−57	39	30	0.40
R caudate		10	19	4	39	0.37
Subcallosal cortex	25	−4	19	−23	27	−0.34
Pre−rTMS > 24 h post−rTMS						
L putamen		−28	8	4	135	0.4
R cerebellum crus 1		35	−66	−37	123	0.78
R cerebellum crus 6		24	−57	−31	38	0.78
R superior lateral occipital cortex		32	−82	20	27	−0.69
L superior lateral occipital cortex	19	−31	−79	20	81	−0.46
L inferior lateral occipital cortex	19	−42	−71	9	27	−0.59
L superior frontal gyrus	10	−36	57	12	66	0.54
L medial frontal cortex	10	−6	60	−12	59	−0.82
L orbitofrontal cortex	47	−17	13	−23	27	0.44
L anterior middle temporal gyrus	21	−58	−11	−20	56	−0.56
R middle temporal gyrus	22	67	−44	1	27	−0.60
R precentral gyrus	6	37	−11	67	36	0.50
R angular gyrus	40	62	−52	37	28	−0.39
R superior parietal lobule	7	21	−55	53	27	−0.39
Anterior cingulate gyrus	24	−4	−6	34	27	0.44
Pre‐rTMS > 1 week post‐rTMS						
L orbitofrontal cortex	47	−39	16	−10	210	−0.43
L superior frontal gyrus	6	−25	27	50	61	0.47
R superior lateral occipital cortex	19	35	−82	34	36	−0.31
L superior lateral occipital cortex	19	−36	−82	29	111	0.33
L inferior lateral occipital cortex		−50	−74	−18	27	0.27
L angular gyrus	40	−55	−49	23	90	−0.32
R posterior supramarginal gyrus		45	−44	18	63	−0.42
L postcentral gyrus	7	−17	−44	56	27	0.32
R pars opercularis	9	45	16	23	27	−0.19
L parietal operculum cortex	40	−50	−33	23	27	0.34
R posterior middle temporal gyrus	21/22	62	−22	−7	54	−0.30
Glx						
Pre‐rTMS > immediate post‐rTMS						
R angular gyrus	22	62	−57	20	210	0.22
L cerebellum crus 1		−20	−66	−34	117	−0.15
L cerebellum 6		−34	−36	−37	54	−0.16
R superior frontal gyrus	8	18	30	42	108	0.22
R middle frontal gyrus		45	38	−10	27	−0.11
L middle frontal gyrus	6	−39	8	61	53	0.19
R posterior inferior frontal gyrus		43	−25	−26	36	−0.13
R precentral gyrus	9	54	08	18	93	0.20
L postcentral gyrus	40	−53	−33	56	67	0.15
Precuneus	31	15	−63	34	81	0.19
L superior lateral occipital cortex	19	−14	−87	45	86	−0.21
L superior lateral occipital cortex	19	−44	−74	45	67	0.24
R insular cortex	13	29	24	04	63	0.17
R posterior middle temporal gyrus	21	70	−38	−15	62	0.22
R posterior middle temporal gyrus	21	59	−25	−10	27	−0.20
R posterior inferior temporal gyrus	20	62	−25	−26	27	−0.09
R thalamus		10	−30	9	27	−0.14
Pre‐rTMS > 24 h post‐rTMS						
R inferior lateral occipital cortex	19	51	−66	−4	338	−0.30
L inferior lateral occipital cortex	19	−47	−82	9	258	−0.36
L superior lateral occipital cortex		−47	−71	26	27	−0.27
L middle temporo‐occipital	21	−64	−52	7	256	−0.35
R posterior middle temporal gyrus		51	−33	−10	108	−0.27
L posterior middle temporal gyrus	21	−61	−14	−10	225	−0.40
R fusiform gyrus	19	29	−85	−12	156	−0.46
L fusiform gyrus	19	−39	−74	−10	72	−0.38
R temporal fusiform cortex		29	−52	−20	54	−0.40
L temporal fusiform cortex	37	−36	−60	−15	45	−0.31
R superior parietal lobule	7	24	−57	59	149	−0.37
L superior parietal lobule	7	−20	−57	64	72	−0.16
L orbitofrontal cortex		−25	22	−15	99	0.34
L superior frontal gyrus	8	−4	54	34	27	−0.25
L middle frontal gyrus	6	−39	5	42	27	−0.13
R cerebellum 4–5		7	−46	−10	57	−0.21
R amygdala		18	−3	−15	54	0.18
R postcentral gyrus	2	40	−22	39	27	0.18
L postcentral gyrus	7	−14	−41	59	42	0.20
R thalamus		2	−3	4	27	0.24
Pre‐rTMS > 1 week post‐rTMS						
L cerebellum 9		−17	−55	−45	156	−0.39
R temporal fusiform cortex	19/37	26	−55	−10	111	−0.40

*Notes*: The columns list (from left to right) regions showing significant differences in rsFC with the stimulation site that are associated with GABA+/Glx concentrations between pre‐ and post‐rTMS visits (uncorrected *p *< .001; cluster‐mass *p *< .05 FDR corrected), the associated BA, peak MNI coordinates of the cluster, cluster voxel size (≥ 25 voxels), and effect size. Effect sizes represent Fisher‐transformed regression coefficients as a ratio of change in rsFC between the stimulation site (seed) and the correlated region per unit change in metabolite concentration. A positive effect size indicates a decrease in rsFC at the post‐rTMS visit, while a negative effect size indicates an increase in rsFC at the post‐rTMS visit. Average change in GABA+ concentration between pre‐rTMS and immediate post‐rTMS = 0.285 i.u., pre‐rTMS and 24 h post‐rTMS = 0.229 i.u., and pre‐rTMS and 1 week post‐rTMS = −0.1775 i.u. Average change in Glx concentration between pre‐rTMS and immediate post‐rTMS = 0.294 i.u., pre‐rTMS and 24 h post‐rTMS = 0.114 i.u., and pre‐rTMS and 1 week post‐rTMS = −0.165 i.u.

Abbreviations: BA, Brodmann area; FDR, false discovery rate; i.u., institutional units; Glx, glutamate + glutamine; GABA+, GABA + macromolecules; L, left hemisphere; MNI, Montreal Neurological Institute; N.S., no significant difference; R, right hemisphere; rsFC, resting‐state functional connectivity; rTMS, repetitive transcranial magnetic stimulation.

**TABLE 6:** Regions showing altered functional connectivity with the posterior cingulate cortex/precuneus related to GABA+ and Glx changes following accelerated rTMS sessions to the visual cortex

		MNI coordinates				
Metabolite/contrast/region	BA	*x*	*y*	*z*	Voxels	Effect size
GABA+						
Pre‐rTMS > immediate post‐rTMS						
R superior frontal gyrus	6	26	0	64	237	−0.86
L orbitofrontal cortex	47	−44	24	−10	30	0.67
R superior lateral occipital cortex	7	15	−68	59	168	−0.74
L cerebellum crus 1		−50	−52	−40	108	−0.68
L cerebellum crus 2		−25	−68	−40	104	−0.44
R paracingulate gyrus	8	7	24	42	72	−0.81
L paracingulate gyrus	9	−9	46	18	66	0.70
L posterior middle temporal gyrus	21	−66	−33	−7	45	0.63
Pre‐rTMS > 24 h post‐rTMS						
R posterior middle temporal gyrus	21	62	−22	−7	27	0.42
L posterior middle temporal gyrus	21	−66	−22	−10	90	0.78
R superior temporal gyrus	22	48	−25	−1	30	0.38
L superior temporal gyrus	38	−50	19	−23	27	−0.53
L cerebellum crus 1		−47	−49	−29	69	−0.80
R frontal operculum cortex	13	48	16	−1	48	0.72
Brainstem		−1	−38	−40	36	−0.41
R precentral gyrus	6	45	0	45	27	0.51
L precentral gyrus	6	−36	−8	64	32	−0.46
L thalamus		−9	−33	4	27	0.34
Pre‐rTMS > 1 week post‐rTMS						
R cerebellum crus 2		40	−52	−40	54	−0.42
L cerebellum crus 2		−50	−52	−48	51	−0.33
L hippocampus	30	−12	−41	1	45	0.39
R superior frontal gyrus	6	10	24	56	27	−0.21
L thalamus		−4	−22	9	27	0.41
R lingual gyrus	30	21	−44	−7	27	−0.36
Glx						
Pre‐rTMS > immediate post‐rTMS						
R paracingulate gyrus	8	5	27	45	680	−0.28
L paracingulate gyrus	9	−9	49	18	36	0.23
R posterior cingulate gyrus	5	2	−41	72	27	−0.15
R anterior cingulate gyrus	24	−6	5	29	66	−0.21
R posterior supramarginal gyrus	40	43	−46	42	105	−0.26
L precentral gyrus	6	−47	−11	31	27	0.09
R angular gyrus		40	−55	34	105	−0.28
R occipital pole	18	24	−96	20	135	0.21
R inferior lateral occipital cortex	18	32	−87	−7	63	0.19
R superior lateral occipital cortex	7	32	−71	56	38	−0.22
L superior lateral occipital cortex		−44	−68	26	57	−0.21
R orbitofrontal cortex	45	51	33	−7	60	0.25
L cerebellum crus 2		−28	−68	−40	36	−0.18
Pre‐rTMS > 24 h post‐rTMS						
R fusiform gyrus		18	−79	−18	144	−0.35
R paracingulate gyrus	32	10	11	39	48	0.35
L paracingulate gyrus	32	−4	27	34	99	0.25
R planum polare	13	51	3	−4	87	0.31
L planum polare	22	−58	−3	−1	36	0.32
R superior lateral occipital cortex	7	18	−68	59	66	−0.17
L superior parietal lobule	7	−28	−46	56	63	−0.29
R superior temporal gyrus	22	51	13	−10	45	0.32
R inferior temporal gyrus	37	56	−52	−18	27	−0.14
R posterior middle temporal gyrus	22	62	−22	−4	57	0.19
Subcallosal cortex		2	13	−4	27	−0.17
Pre‐rTMS > 1 week post‐rTMS						
R accumbens		10	13	−10	108	0.43
L cerebellum crus 1 and 2		−47	−49	−40	90	−0.42
R superior lateral occipital cortex	7	35	−66	59	72	−0.39
R lingual gyrus	18	10	−90	−10	54	0.21
R inferior temporo‐occipital	37	62	−52	−18	54	−0.22
R anterior supramarginal gyrus	2	59	−22	31	54	−0.37
L frontal pole	10	−17	63	−18	28	−0.31
L middle frontal gyrus	8	−42	35	34	27	0.40
Anterior cingulate gyrus	32	2	38	15	27	0.14
L fusiform gyrus		−31	−66	−15	27	−0.27

*Notes*: The columns list (from left to right) regions showing significant differences in rsFC with the posterior cingulate cortex/precuneus that are associated with GABA+/Glx concentrations between pre‐ and post‐rTMS visits (uncorrected *p *< .001; cluster‐mass *p *< .05 FDR corrected), the associated BA, peak MNI coordinates of the cluster, cluster voxel size (≥25 voxels), and effect size. Effect sizes represent Fisher‐transformed regression coefficients as a ratio of change in rsFC between the posterior cingulate cortex/precuneus (seed) and the correlated region per unit change in metabolite concentration. A positive effect size indicates a decrease in rsFC at the post‐rTMS visit, while a negative effect size indicates an increase in rsFC at the post‐rTMS visit. Average change in GABA+ concentration between pre‐rTMS and immediate post‐rTMS = 0.285 i.u., pre‐rTMS and 24 h post‐rTMS = 0.229 i.u., and pre‐rTMS and 1 week post‐rTMS = −0.1775 i.u. Average change in Glx concentration between pre‐rTMS and immediate post‐rTMS = 0.294 i.u., pre‐rTMS and 24 h post‐rTMS = 0.114 i.u., and pre‐rTMS and 1 week post‐rTMS = −0.165 i.u.

Abbreviations: BA, Brodmann area; FDR, false discovery rate; i.u., institutional units; Glx, glutamate + glutamine; GABA+, GABA + macromolecules; L, left hemisphere; MNI, Montreal Neurological Institute; N.S., no significant difference; R, right hemisphere; rsFC, resting‐state functional connectivity; rTMS, repetitive transcranial magnetic stimulation.

The effect size values in Tables 3 and 6 represent connectivity strength as a ratio of change in rsFC between the seed and correlated cluster per unit change in metabolite concentration. The average change in metabolite concentration at the stimulation site (average pre‐rTMS concentration minus post‐rTMS concentration) corresponding to the rTMS protocol is provided in the corresponding Table captions.

## 4 DISCUSSION

We report a combination of interdisciplinary methods to further our understanding of rTMS‐induced effects at the visual cortex and associated pathways. Initial data demonstrate unexpected differential rsFC changes in nodes associated with the stimulation site (V1) and indirectly with the DMN following two distinct low‐frequency (1 Hz) rTMS protocols. A single 20 min session produced delayed strong changes in rsFC across cortical and subcortical regions. Surprisingly, five accelerated 20 min sessions of rTMS produced weak and relatively insignificant changes in rsFC. We further observed that rsFC was associated with changes in GABA+ and Glx concentrations with both rTMS protocols, and these changes to rsFC fluctuated between post‐rTMS visits. This study demonstrates that 1 Hz rTMS modulates rsFC with regions distal to the stimulation site and with regions that are not usually functionally connected to the seed ROIs pre‐rTMS.

### 4.1 Effect of low‐frequency rTMS to the visual cortex on intrinsic functional connectivity

The resting‐state visual network consists of primary visual and extrastriate cortices with additional functional connectivity with the motor network (van den Heuvel et al., 2009). A single 20 min session of 1 Hz rTMS to the visual cortex modulated rsFC (correlated and anticorrelated) to cortical regions outside of the usual visual network (Table 1), including nodes of the DMN (e.g., precuneus and middle frontal gyrus), and subcortical regions (e.g., cerebellum and thalamus). The DMN consists of overlapping posterior cingulate cortex/precuneus, anterior cingulate cortex, frontal, parietal, and temporal regions (M. D. Greicius et al., 2003; Laird et al., 2009). Although we did not directly stimulate nodes of the DMN, the posterior cingulate cortex/precuneus also showed modified rsFC to regions outside of the DMN post‐rTMS, for example, the basal ganglia and cerebellum (Table 2). Increasing evidence suggests that specific cerebellar regions are integral to the DMN (Brissenden et al., 2016; Buckner et al., 2011). The cerebellum is rarely reported as a DMN node, partly as a result of studies not acquiring whole‐brain data and/or employing insensitive acquisition or analysis methods. Identifying subcortical effects in the present study may attribute to ME‐ICA sensitivity. These subcortical effects are reported as unobservable with single‐echo rsfMRI at the current sample size and with alternative denoising techniques where effects are obscured by greater amounts of non‐BOLD noise (e.g., low functional contrast‐to‐noise due to cerebral spinal fluid and blood flow pulsatility; Kundu et al., 2012; Lombardo et al., 2016).

Notably, single‐session rTMS effects did not emerge until 1 h post‐rTMS for both seed ROIs (Tables 1 and 2). Delayed rsFC changes may suggest that immediate post‐rTMS effects are too diffuse and weak to be detected or that there is greater interindividual variability resulting in nonsignificant findings. To investigate weaker effects, we used a less conservative voxel‐level threshold (*p* < .01), yet we continued to observe no significant rsFC changes between pre‐ and immediate post‐rTMS visits. Exploring individual‐level rsFC maps to assess variability also revealed little change in connectivity values for either seed ROIs between pre‐ and immediately post‐rTMS visits. The confirmed absence of significant rsFC changes immediately post‐rTMS suggests that other underlying neural factors are at play resulting in delayed changes following a single rTMS session or perhaps that rsfMRI may be insensitive to initial subtle changes. However, ME‐ICA is considered to be more sensitive to slower emerging changes with TMS than previous conventional methods using single‐echo rsfMRI and alternative denoising techniques (e.g., band‐pass filtering) that are unlikely to capture these effects (Evans et al., 2015).

It is important to note that rsFC changes following a single session of rTMS outlast those measured by other neurotechniques that have recorded shorter aftereffects. Our knowledge of rTMS aftereffects has relied heavily on electroencephalography (EEG) recordings. In the case of low‐frequency rTMS to the visual cortex, EEG recordings following 10−20 min of 1 Hz rTMS have established aftereffects lasting in the range of 10−40 min (for a review, see Thut & Pasucal‐Leone, 2010). Accordingly, we did not expect rsFC changes to persist at 1 h post‐rTMS following a single rTMS session and certainly not that these effects would only begin to arise and/or stabilize at the 1 h mark. This finding critically highlights that our understanding of noninvasive brain stimulation has been highly dependent and limited on the tool of measure. Accelerated theta burst rTMS (TBS) has also been shown to cause changes in excitability (measured by motor evoked potentials) that are incongruent with changes in motor network rsFC (Nettekoven et al., 2014). Collectively, these findings suggest that aftereffects observed using electrophysiological measures may capture different mechanisms compared to functional connectivity. Alternatively, rsfMRI may be less sensitive to immediate post‐rTMS effects and instead show delayed network changes, whereas EEG may be more sensitive to initial rTMS‐induced changes, or it may be due to temporal resolution differences between the methods. In short, rsfMRI rTMS aftereffects appear to persist longer than those measured by EEG.

We observed a rather unexpected finding of null effects following five accelerated 20 min sessions of rTMS on rsFC. We predicted either an inversion of effects compared with single rTMS as is observed with longer trains of stimulation (e.g., Gamboa et al., 2010) or a strengthening of effects (e.g., Nettekoven et al., 2014). One possible explanation for this null finding is the presence of large interindividual variability in the TMS response (Ridding & Ziemann, 2010; Stewart et al., 2001). Again, investigation of individual‐level connectivity values following accelerated rTMS showed very little change in rsFC across visits and participants. To test for weaker effects, we repeated analyses with a less conservative uncorrected voxel‐level threshold (*p* < .01) and did in fact observe weak and diffuse differences in rsFC following accelerated rTMS (Tables S2 and S3). These diffuse changes encompassed a large number of brain regions (clusters > 1000 voxels) that were detected only at 24 h post‐rTMS for effects associated with the stimulation site seed, and at 1 week post‐rTMS for effects associated with the posterior cingulate cortex/precuneus seed. The continued absence of immediate post‐rTMS effects suggests that these weak accelerated rTMS effects do not become stable and/or arise until at least 24 h, yet they persist for at least 1 week.

An alternative consideration for accelerated rTMS effects relates to stimulation‐induced metaplasticity and the ability of functional cortical networks to maintain neuronal activity within a dynamic range (Bocci et al., 2014; Lang et al., 2004). It could be, at least in terms of rsFC, that the brain becomes resistant to change and the dose‐dependent effects of accelerated rTMS occur as a compensatory mechanism to maintain homeostasis in healthy individuals. Support for this resistance effect comes from the strong rsFC changes observed following just a single rTMS session that are not seen with subsequent stimulation. Gamboa and colleagues (2011, 2010) have also observed suppression of aftereffects following accelerated TBS sessions to the motor cortex within 1 day that were dependent on the TBS protocol and the timing of intervals between sessions. A commonly proposed concept for these compensatory aftereffects with multiple rTMS sessions is the Bienenstock–Cooper–Munro theory (Bienenstock et al., 1982). Hebbian synaptic plasticity enables a continuous unidirectional change in network excitability following stimulation that would essentially destabilize a neural system (Bocci et al., 2014). The Bienenstock–Cooper–Munro theory proposes that long‐term potentiation (LTP) induced after stimulation favors the induction of long‐term depression (LTD) with subsequent stimulation, thereby preventing an excessive buildup of LTP or LTD. This mechanism regulates intrinsic excitability, and ensures stable neuronal activity through dynamic modification of LTP and LTD thresholds. Conversely, a strengthened effect has been recorded with multi‐day and accelerated stimulation protocols in patient populations (for a review, see Goldsworthy et al., 2014; Rafique et al., 2016) and healthy individuals (Bastani & Jaberzadeh, 2014; Nettekoven et al., 2014). Contradictory findings across studies pertain to differences in cohorts, stimulation protocols, intervals between multiple stimulation sessions, site of stimulation, and the neuroimaging techniques employed to measure aftereffects.

### 4.2 Relationship between GABA+ and Glx with functional connectivity changes

We have previously observed a reduction in MRS‐measured GABA+ at the visual cortex with the same accelerated rTMS protocol (Rafique & Steeves, 2020), which demonstrates that the compensatory “restorative” effect at the functional connectivity level is not consistent across biomarkers. Our distinct findings further highlight sensitivity differences between neuroimaging methods in measuring aftereffects. If we consider the relationship between GABA+/Glx concentrations and rsFC changes following both rTMS protocols, we observe significant widespread changes associated with both seed ROIs that persist until at least the last follow‐up visit (Tables 3, 4, 5, 6). The influence of other underlying neural mechanisms on network connectivity beyond BOLD signal should be considered to achieve a more complete understanding of neuromodulation protocols. The interaction of rsFC with glutamatergic (precursor to GABA) and GABAergic systems may be a more appropriate indicator of TMS effects. Functional connectivity is influenced by cortical network oscillations in the gamma frequency range (Cabral et al., 2011). In the visual cortex, gamma frequency oscillations are positively related with MRS‐measured GABA and inversely correlated with the magnitude of the BOLD response (Muthukumaraswamy et al., 2009). Synchronized neural oscillations in cortical networks are more specifically mediated by postsynaptic GABA_A_ receptors (for a review, see Gonzalez‐Burgos & Lewis, 2008). MRS is thought to be insensitive to synaptic activity, but instead measures the total GABA concentration within the voxel and reflects extrasynaptic GABAergic tone (Stagg et al., 2011). Spontaneous neurotransmitter release at synapses additionally occurs in the absence of neuronal spikes or action potentials and signifies an important component of spontaneous fluctuations (for a review, see Kavalali, 2015). Both rsFC and MRS are indirect measures differentially sensitive to neurotransmitter changes.

Glx concentrations were not correlated with rsFC changes with the posterior cingulate gyrus/precuneus following a single session of rTMS (Table 4). Following accelerated rTMS, both GABA+ and Glx changes modified rsFC with the posterior cingulate gyrus/precuneus (Table 6). Prior work demonstrates that both glutamate and GABA are significantly associated with DMN activity (Hu et al., 2013; Kapogiannis et al., 2013). Our findings imply that Glx is not sufficiently altered at the visual cortex following a single session of 1 Hz rTMS to modify indirect rsFC changes with the posterior cingulate gyrus/precuneus. Both GABA+ and Glx concentrations were associated with rsFC changes with the stimulation site (V1) following single and accelerated rTMS sessions. This is consistent with our previous finding where only accelerated rTMS had greater potential to influence plasticity by significantly impacting GABA concentrations (Rafique & Steeves, 2020). Correspondingly, we observed more widespread rsFC changes following accelerated rTMS than single‐session when taking into account metabolite changes.

### 4.3 Implications of low‐frequency rTMS on functional connectivity associated with the visual cortex

We aimed to address the lack of literature examining the potential of rTMS to modulate widespread functional connectivity associated with the visual cortex. While much work has been done in nonvisual brain regions, there have been limited investigations of TMS‐induced neural effects in visual pathways and a shortage of whole‐brain functional connectivity studies. These findings have implications for understanding underlying neural effects of low‐frequency rTMS to the visual cortex for therapeutic application in visual‐related disorders and in experimental science where TMS is often employed to map visual cortical connectivity, infer functionality, and measure causal relationships between regions.

It is beyond the scope of this study to provide a comprehensive explanation for each region connected with the seed ROIs, the associated change in connectivity, and describe the implication on function for each region involved. However, several factors aid in interpreting the widespread and dose‐dependent findings that shape our results. We have previously shown that low‐frequency rTMS does not simply “inhibit” activity (as is generalized) at the stimulation site or interconnected regions, but that it attempts to restore previously imbalanced cortical activity (Rafique et al., 2016). Studies also demonstrate that low‐frequency rTMS to visual processing regions does inhibit activity in interconnected regions when they are involved in similar category/function‐selective processing (e.g., Rafique et al., 2018; Solomon‐Harris et al., 2016). Additionally, the partial coherency between regions based on fMRI time‐series depends on the anatomical distance between regions (Salvador et al., 2005). Long‐distance or remote intrahemispheric connections (greater than 7 cm; e.g., prefrontal and parietal cortex) are mediated structurally by white matter tracts and show greater functional connectivity at low frequencies than at frequencies greater than 0.3 Hz. Similarly, bilaterally homologous brain regions are strongly and symmetrically connected with greater functional connectivity at low frequencies. Local or short‐distance connections (e.g., distinct dorsal and ventral paths in the posterior cortex) show high‐frequency connectivity but are generally stronger than long‐distance connectivity. When white matter tracts are absent, functional connectivity is significantly reduced for high‐ compared to low‐frequency bands. Moreover, it has been shown that dynamic interactions at different natural frequencies (the dominant oscillation rate) reflect specific intrinsic properties of discrete cortical regions and their interconnections. Neural activity in distinct frequency bands plays a distinct role in perception, motion, and cognition (Basar et al., 2000, 2001). Correlations are observed between alpha rhythms in specific occipital, parietal, and temporal regions; beta rhythms in certain frontal, occipital, orbital, and parietal regions; and between gamma rhythms and frontal cortex activity—although significant variations in topography occur depending on the task (Gomez et al., 2006; Gomez‐Herrero et al., 2008; Feige et al., 2005; Laufs et al., 2003; Mantini et al., 2007). These connectivity patterns are constant within the same subject, although they exhibit intersubject variations (Cona et al., 2011). TMS is observed to decrease function–structure correlation in each frequency. TMS effects propagate towards other regions or modify intrinsic rhythms, stimulating several connected regions. The resultant TMS‐induced response consists of strong oscillations at the natural frequency of the stimulated area and weaker fluctuations at the natural frequency of remote regions indirectly engaged through brain connections, generating a wide convoluted pattern of frequency‐influenced interactions in the whole‐brain network (Amico et al., 2017; Rosanova et al., 2009). A return to baseline is hypothesized to depend on the temporal duration of functional activation of the elicited area and the magnitude of its structural connectivity pattern (Amico et al., 2017). Finally, the changing involvement of regions between pre‐ and post‐rTMS visits or the differential association with metabolites is further influenced by cortical excitability. Regions demonstrate distinct excitability thresholds (Stewart et al., 2001; Stokes et al., 2005), and variations in cytoarchitecture and connectivity within subregions (van den Heuvel et al., 2015). In summary, the relationship between visual cortical regions and remote regions following rTMS is dependent on functional activation at specific resonant frequencies and excitability thresholds, structural and/or functional coupling, and anatomical architecture of the specific brain region.

We do not intend to predict effects in pathophysiology coexisting with impaired functional state and altered responsiveness to rTMS (e.g., Antal et al., 2008; Rafique et al., 2015). We know from patient populations that accelerated rTMS has a stronger cumulative effect than conventional single sessions applied over consecutive days (for a review, see Goldsworthy et al., 2015). It is highly plausible that these promising results obtained in patient populations are owed to impaired systems (e.g., inability to maintain homeostasis, and impaired metaplasticity), and that the magnitude and direction of neuromodulated effects induced by rTMS will differ considerably from healthy subjects. It is, however, necessary to first investigate rTMS‐induced responses in healthy controls in the absence of pathophysiology for a number of reasons. Developing protocols requires comparative data to understand the typical connectivity response to stimulation so as not to worsen pathology. Deviations in response between patient and healthy populations also provide considerable insight into disease mechanisms and can highlight disease‐driven biomarkers. Additionally, it is crucial to consider inadvertently induced perceptual, neurobiochemical, and behavioral adverse effects. The potential for adverse effects remains underinvestigated despite the increasing therapeutic use of noninvasive brain stimulation in greater doses in several clinical conditions. Adverse effects are particularly possible in patient populations due to the unpredictability of disease (Maeda et al., 2000; Wassermann, 2002). There is an even greater likelihood of adverse effects with increasing stimulation doses (e.g., multiple sessions across consecutive days) as is used in patients. For example, the DMN is involved in cognitive function associated with intrinsic processing and external inputs (Fox et al., 2005) and may become impaired with greater stimulation doses. Other regions affected by rTMS in our study are implicated in auditory function (e.g., planum polare, insular cortex), executive control (e.g., prefrontal and cingulate cortices) (for a review, see Beckman et al., 2005), as well as attention (e.g., middle temporal and prefrontal regions; Fox et al., 2005; Laufs et al., 2003). Although we observed considerable widespread rsFC changes, our protocols did not cause measurable or perceptual deficits in visual or cognitive function, nor did participants report significant adverse effects (Rafique & Steeves, 2020). Future work is needed to investigate how overall rsFC changes translate to an improvement or decline in performance. The direction of effect (correlated or anticorrelated) is another aspect requiring attention when considering desired neuromodulation effects given that a protocol may worsen an already aberrant connection between regions. Abnormalities in correlated and anticorrelated networks are observed in a number of neuropsychiatric disorders (M. Greicius, 2008; Mulders et al., 2015; Whitfield‐Gabrieli & Ford, 2012). In the present study, we observed a shift from correlated to anticorrelated rsFC and vice versa in some regions following rTMS, likely to maintain a somewhat dynamic and functionally organized system.

Knowledge of differential effects for a variety of stimulation protocols will enable modulation of mechanisms suited to a greater number of disorders presenting with variable pathophysiology. This would advance the development of therapies in terms of the most efficient protocol combining optimal effects with minimal stimulation time (e.g., stimulation over days rather than weeks to improve patient compliance). Identifying functionally interconnected nodes is important if one wishes to target specific networks. Lesion location mapping studies suggest stimulating nodes functionally connected to the lesion since it will propagate to the connected lesion site (Boes et al., 2015). Not only are remote nodes interconnected to the damaged tissue targeted but regions displaying aberrant activity close to the lesion are also targeted (e.g., Boes et al., 2015), thereby targeting multiple nodes at once. Alternatively, to target a node/region that may lie outside the stimulation depth parameters of TMS (Zangen et al., 2005), one can target accessible distal nodes interconnected with the region(s) of interest implicated in specific disorders. Whether visual cortical stimulation is applied to patient or healthy populations using a combination of techniques as in the present study would enable a more sensitive and complete representation of effects.

### 4.4 Methodological considerations

We employed a smaller sample size for this proof‐of‐concept study. However, we used conservative statistical thresholds and employed nonparametric methods to limit false positive findings. Additionally, we used a within‐subject design (single and accelerated groups) to decrease interindividual variability of TMS effects and to increase statistical power. Despite the small sample size, we observed strong changes in rsFC with minimal interindividual variability across visits. ME‐ICA also substantially improves effect size estimates and statistical power with traditional small sample sizes in fMRI studies by specifically addressing problems related to non‐BOLD artefact variability (Lombardo et al., 2016), while remaining conservative in the cut‐off for retaining BOLD signal components (Evans et al., 2015).

Other factors to consider included methodological and practical limitations and careful coordination and timing of events. We employed strict inclusion criteria to minimize external influences on metabolite receptors and TMS mechanisms (for a full description, see ‘‘Participants’’ section in Rafique & Steeves, 2020). We also had to ensure that the timing of acquisition would capture immediate post‐rTMS effects and prevent diluting of effects that could occur with too long an acquisition protocol. Particularly with the longer accelerated rTMS sessions, it was necessary to develop a protocol minimizing participant fatigue and discomfort that may confound data. Additionally, we had to consider MRS associated constraints (see ‘‘Considerations’’ section in Rafique & Steeves, 2020). Changes in metabolite concentrations were obtained from the stimulation site at the visual cortex (V1). Therefore, metabolite changes associated with rsFC changes with the posterior cingulate cortex/precuneus are with reference to metabolite values obtained from the stimulation site. Given that the stimulation site shows significant correlations to the posterior cingulate cortex/precuneus and other nodes in the DMN, it is expected that metabolite changes would be relayed to interconnected nodes. When considering network effects associated with the posterior cingulate cortex/precuneus, the indirect metabolite influence is essential in making inferences. We acknowledge that metabolite concentration changes at the posterior cingulate cortex/precuneus seed will indeed be different; however, we can only obtain single‐voxel MRS acquisition (see ‘‘Considerations’’ section in Rafique & Steeves, 2020). We can obtain direct measures in a separate experiment, which would require repeating the full experiment with all follow‐up visits using an MRS voxel at the posterior cingulate cortex/precuneus. This would have the added limitation that the functional state of the individual might be different.

Our understanding of 1 Hz rTMS aftereffects is limited by our follow‐up visit time intervals. We observed changes that likely continue well past our last time points and require further investigation to determine when changes stabilize and return to baseline following rTMS (e.g., including a 2‐week follow‐up post‐accelerated rTMS). Our follow‐up visits for the two groups were guided by previous literature. It is apparent from our findings that previous literature provides a limited understanding of aftereffects due to selectively sensitive neurotechniques. Moreover, to accurately determine whether accelerated rTMS induces a restorative change (i.e., a relatively homeostatic response) in rsFC in healthy controls, rsfMRI would need to be repeated following each consecutive stimulation session. Repeating rsfMRI would require longer breaks between stimulation sessions to allow for MRI set‐up and so forth, which may produce dissimilar results by using longer intervals (e.g., Gamboa et al., 2011; Goldsworthy et al., 2015). Longer breaks and added fMRI acquisitions would be extremely taxing for participants and may introduce confounds. Finally, our findings are limited to effects produced by 1 Hz rTMS at 100% PT to the visual cortex. Previous studies demonstrate that the magnitude and direction of effects are highly dependent on stimulation intensity, that is, sub‐ and supra‐PT stimulation (Di Lazarro et al., 1998; U. L. F. Ziemann et al., 1996).

We did not employ a sham condition as our study was concerned with the methodological aspects of rTMS protocols and their effects on V1 associated rsFC. The efficacy of TMS versus control site/sham is well established from an extensive range of studies in a variety of populations and brain regions. Sham stimulation itself presents with significant limitations since it can induce changes in neural activity through weak stimulation, clicking noises, or the tapping sensation of stimulation pulses. Sham coils induce low strength electric fields up to 25.3% of their respective active values (J. E. Smith & Peterchev, 2018). With the Magstim active coil, the center has the strongest stimulation. However, the Magstim sham coil produces electric fields with stronger stimulation in the periphery (3−7 cm from the center). Additionally, participants are aware that the sensation and clicking noise with sham stimulation is different to active stimulation, thus unblinding participants (Arana et al., 2008; Duecker & Sack, 2015; Jung et al., 2016). Given the extensive whole‐brain effects of rTMS and changeability of involved nodes, isolating a “control” site would not be feasible since it can be directly or indirectly connected to the stimulation site of interest.

## 5 CONCLUSION

The findings from the present study reveal that focal disruption to the visual cortex with low‐frequency rTMS alters neuroplasticity and the spatial topography of the whole‐brain network. These results have important implications for developing therapeutic protocols for visual‐related disorders in that single‐session rTMS to the visual cortex may be more effective than accelerated rTMS in targeting network connectivity depending on the pathophysiology and interactions with neurotransmitter levels. ME fMRI provides an important tool to investigate longer‐lasting TMS‐induced aftereffects across multiple networks over conventional and differentially sensitive methods such as EEG that demonstrate shorter duration aftereffects. In summary, we demonstrate the value and necessity in employing combined neuroimaging techniques with neuromodulation for a more complete understanding of TMS‐induced effects, as well as describing key considerations in experimental design. We further provide data to inform future research, and ultimately provide a basic foundation of crucial work to build on. These methods/analysis techniques can be readily altered to suit the question at hand, including exploring other networks of interest, and using follow‐up time points that allow direct comparison of protocols.

## CONFLICT OF INTEREST

The authors declare no conflict of interest.

## AUTHOR CONTRIBUTIONS

*Conceptualization*: Sara A. Rafique. *Methodology*: Sara A. Rafique and Jennifer K. E. Steeves. *Investigation*: Sara A. Rafique. *Project administration*: Sara A. Rafique. *Formal analysis*: Sara A. Rafique. *Resources*: Sara A. Rafique. *Writing ‐ original draft*: Sara A. Rafique. *Writing ‐ review & editing*: Jennifer K. E. Steeves. *Supervision*: Jennifer K. E. Steeves. *Funding acquisition*: Sara A. Rafique and Jennifer K. E. Steeves.

### PEER REVIEW

The peer review history for this article is available at https://publons.com/publon/10.1002/brb3.2491

## Supporting information

Supporting information

## Data Availability

Data available on request due to privacy/ethical restrictions.
