# Supplementary material for: Modulating intrinsic functional connectivity with visual cortex using low‐frequency repetitive transcranial magnetic stimulation
Source: Brain Behav. 2022 Jan 20;12(2):e2491. doi: 10.1002/brb3.2491 (PMC8865167; doi:10.1002/brb3.2491)
Supplement: Supplementary file 1 — Supporting information [file BRB3-12-e2491-s001.pdf]

SUPPORTING INFORMATION

Modulating intrinsic functional connectivity with visual cortex using low-frequency repetitive transcranial magnetic stimulation

Sara A. Rafique and Jennifer K. E. Steeves

Department of Psychology and Centre for Vision Research, York University, Toronto, Canada

Table S1. Individual PT and MNI coordinates for the rTMS target site at the visual cortex

rTMS group/ Participant #	PT (%)	MNI coordinates		
		x	y	z
**Single rTMS session**				
1	71	1	-83	13
2	79	1	-79	18
3	70	0	-84	16
4	70	0	-84	7
5	51	0	-83	8
6	69	-1	-84	11
7	74	1	-75	23
8	65	4	-82	13
**Accelerated rTMS sessions**				
9	76	-5	-83	9
10	77	2	-79	15
11	77	2	-82	12
12	67	-1	-83	15
13	49	1	-80	20
14	85	1	-76	19
15	80	1	-78	20
16	74	1	-85	14

Note. The columns list (from left to right) each participant (identified numerically) and their associated rTMS group, PT (intensity/power at which rTMS was delivered), and peak MNI coordinates where 10 mm radius sphere seed ROIs were centered. PT = phosphene threshold; rTMS = repetitive transcranial magnetic stimulation; MNI = Montreal Neurological Institute; ROI = region-of-interest.

Table S2. Regions showing weak changes in functional connectivity with the visual cortex (stimulation site) following accelerated rTMS sessions

Contrast/ Region	MNI coordinates			Voxels	Effect size
	x	y	z		
**Pre-rTMS > immediate post-rTMS**					
N.S.					
**Pre-rTMS > 24 hr post-rTMS**					
Cluster 1	-12	-63	67	3511	0.20[b]
Precuneus				1727	
L superior parietal lobule				491	
R superior lateral occipital cortex				12	
L superior lateral occipital cortex				430	
R postcentral gyrus				10	
L postcentral gyrus				184	
**Pre-rTMS > 1-week post-rTMS**					
N.S.					

Note. The columns list (from left to right) regions within the cluster showing significant differences in rsFC with the stimulation site between pre- and post-rTMS visits (uncorrected $p < .01$; cluster-mass $p < .05$ FDR corrected), peak MNI coordinates of the cluster, cluster voxel size (≥ 10 voxels), and effect size of the cluster. Effect sizes represent the average difference in Fisher-transformed correlation coefficients between visits (pre-TMS visit minus the post-rTMS visit) for the stimulation site (seed) and the correlated region. A positive effect size indicates a decrease in rsFC at the post-rTMS visit. rTMS = repetitive transcranial magnetic stimulation; MNI = Montreal Neurological Institute; rsFC = resting-state functional connectivity; FDR = false discovery rate; R = right hemisphere; L = left hemisphere; N.S. = no significant difference.
[b]anticorrelated post-rTMS.

Table S3. Regions showing weak changes in functional connectivity with the posterior cingulate cortex/precuneus following accelerated rTMS sessions to the visual cortex

Contrast/ Region	MNI coordinates			Voxels	Effect size
	x	y	z		
**Pre-rTMS > immediate post-rTMS**					
N.S.					
**Pre-rTMS > 24 hr post-rTMS**					
N.S.					
**Pre-rTMS > 1-week post-rTMS**					
Cluster 1	54	-41	1	4364	0.20[b]
R middle temporo-occipital				1597	
R posterior middle temporal gyrus				159	
R posterior superior temporal gyrus				136	
R angular gyrus				715	
R posterior supramarginal gyrus				207	
R temporal fusiform cortex				32	
R superior lateral occipital cortex				15	
R inferior lateral occipital cortex				10	

Note. The columns list (from left to right) regions within the cluster showing significant differences in average rsFC with the posterior cingulate cortex/precuneus between pre- and post-rTMS visits (uncorrected $p < .01$; cluster-mass $p < .05$ FDR corrected), peak MNI coordinates of the cluster, cluster voxel size (≥ 10 voxels), and effect size of the cluster. Effect sizes represent the average difference in Fisher-transformed correlation coefficients between visits (pre-TMS visit minus the post-rTMS visit) for the posterior cingulate cortex/precuneus (seed) and the correlated region. A positive effect size indicates a decrease in rsFC at the post-rTMS visit. rTMS = repetitive transcranial magnetic stimulation; MNI = Montreal Neurological Institute; rsFC = resting-state functional connectivity; FDR = false discovery rate; R = right hemisphere; N.S. = no significant difference. [b]anticorrelated post-rTMS.
